# Supplementary figures and images for: Pam16 and Pam18 were repurposed during Trypanosoma brucei evolution to regulate the replication of mitochondrial DNA
Source: PLoS Biol. 2024 Aug 15;22(8):e3002449. doi: 10.1371/journal.pbio.3002449 (PMC11349236; doi:10.1371/journal.pbio.3002449)

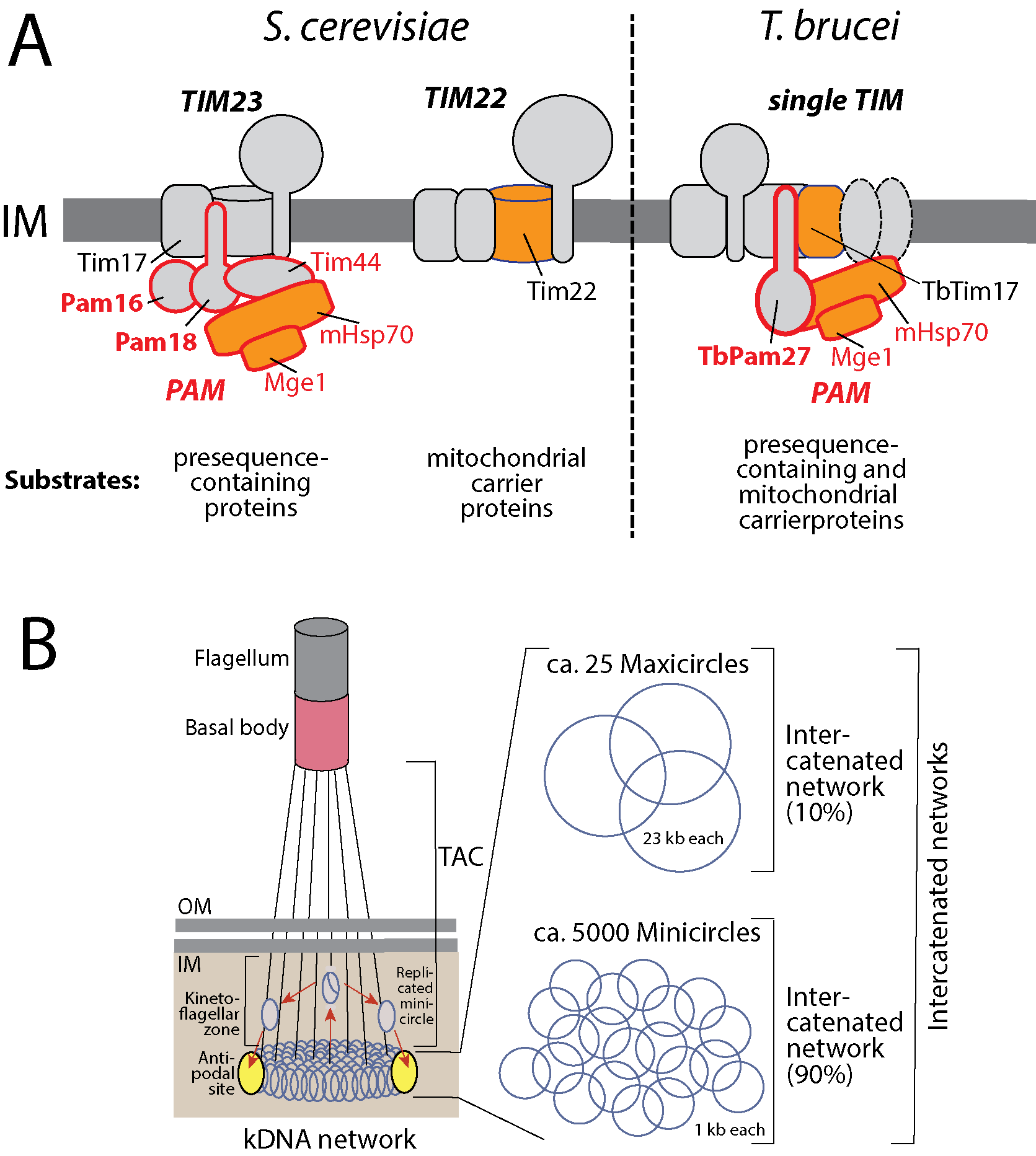

Supplement: S1 Fig — (A) Schematic depiction of the yeast TIM23 and TIM22 complexes (left) and the single trypanosomal TIM complex (right). In the trypanosomal TIM complex, the subunits specifically associated with presequence pathway are indicated in broken lines. Subunits of the presequence-associated import motor (PAM) are indicated in bold red lines. Unique and homologous subunits between the 2 species are indicated in gray and orange, respectively. The non-homologous J domain proteins Pam18/16 and TbPam27 are indicated in bold. (B) Organization of the single unit kDNA of T. brucei. The kDNA is a disk consisting of an intercalated network of maxi- and minicircles, which is physically connected, across the outer and the inner membrane, with the basal body of the flagellum via the tripartite attachment complex (TAC). Minicircles are replicated via theta structures after they have been released from the center of the network into the kinetoflagellar zone. After replication, they are reattached to the kDNA disk at the antipodal sites. Maxicircles in contrast are replicated while remaining attached to the kDNA disk. (TIF) [file pbio.3002449.s001.tif]

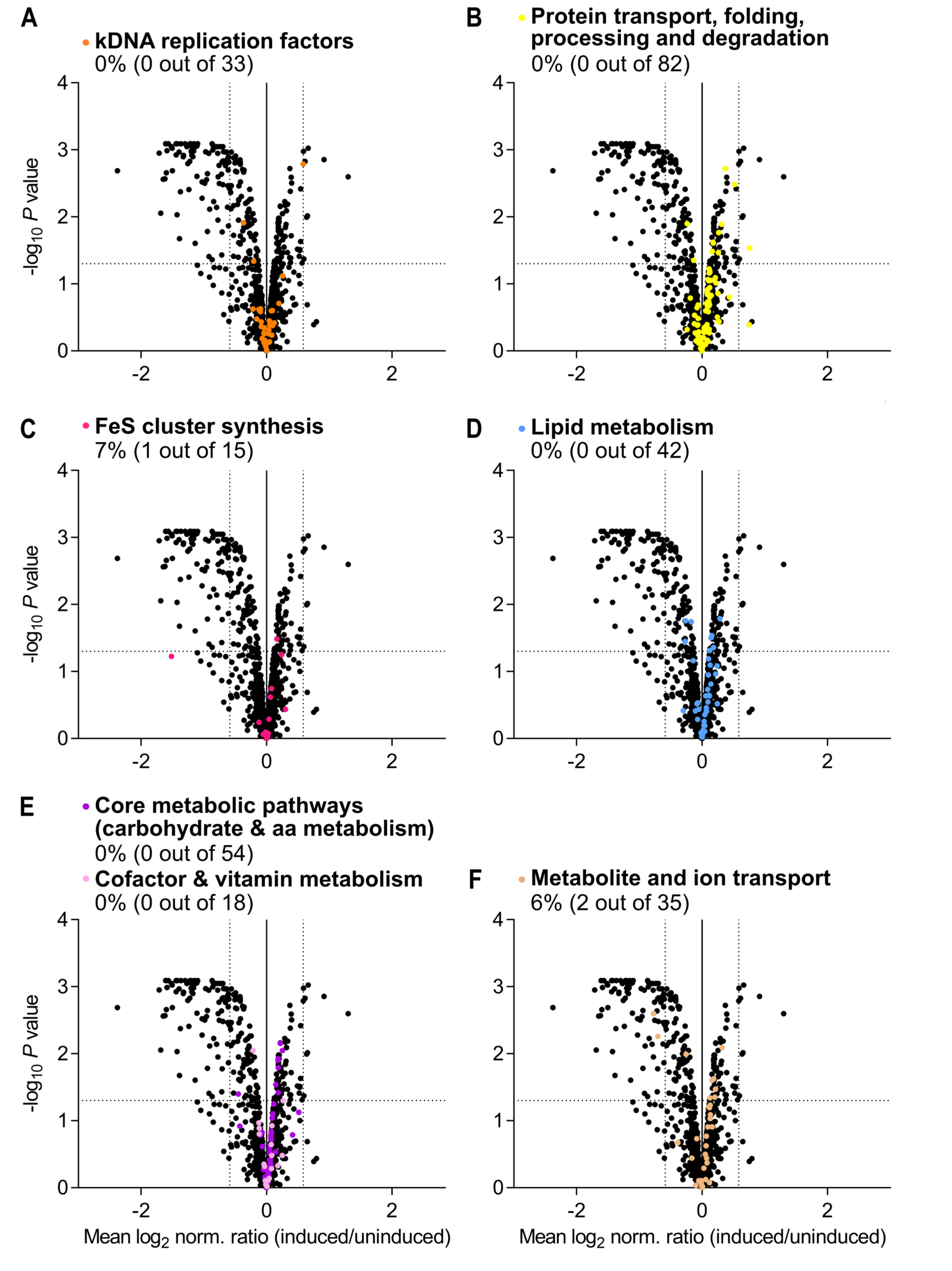

Supplement: S2 Fig — Mitochondria-enriched fractions of uninduced and 4 days induced TbPam18 RNAi cells were analyzed by SILAC-based quantitative mass spectrometry (same data set as in Fig 1B). (A–F) The indicated subgroups of mitochondrial proteins are highlighted in the indicated colors. The number of all more than 1.5-fold depleted proteins and the total number of all detected proteins for each subgroup are indicated in parentheses at the top of each panel. Numerical data for panel (A) to (F) are available in S1 Table. (TIF) [file pbio.3002449.s002.tif]

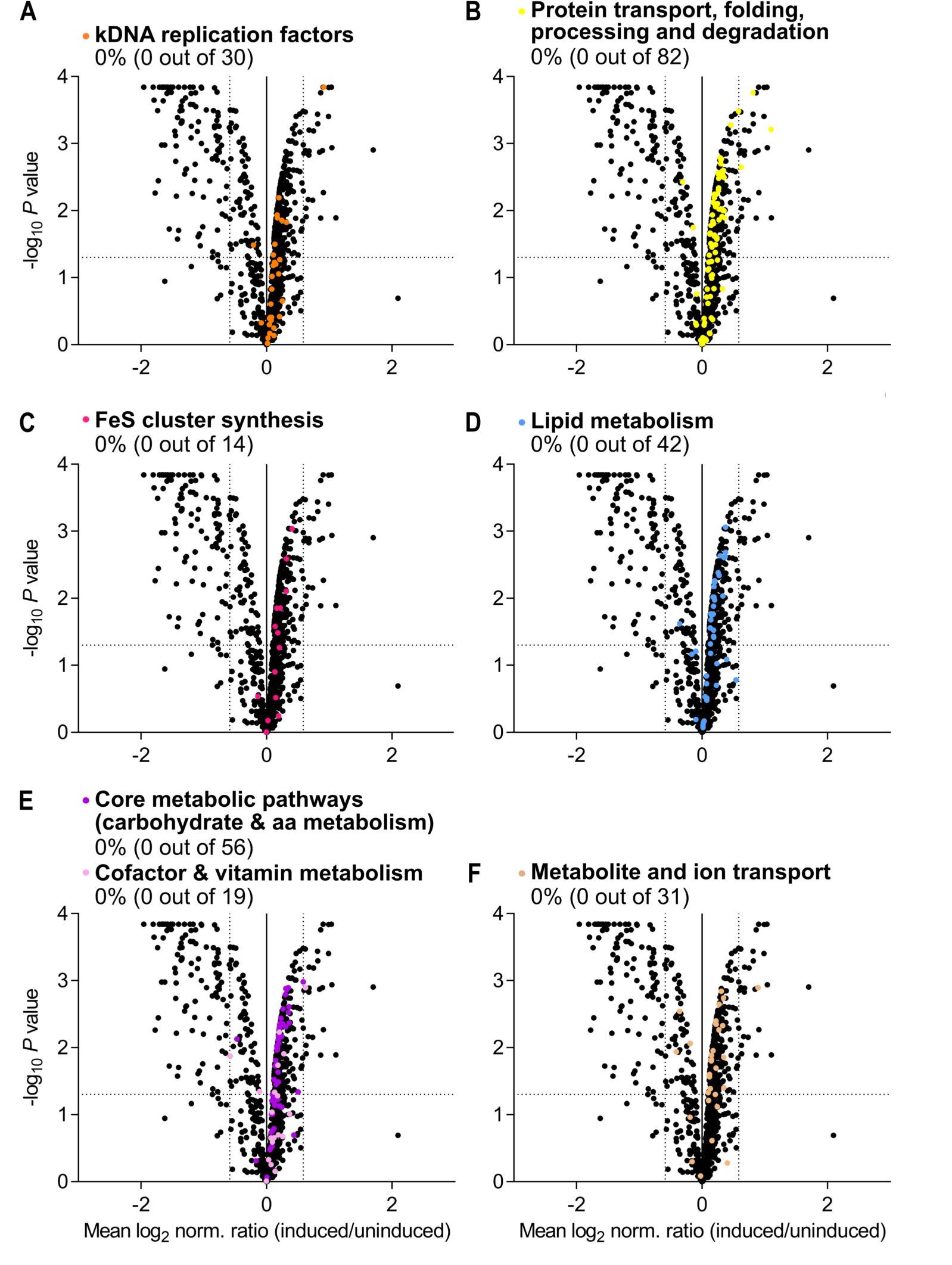

Supplement: S3 Fig — Mitochondria-enriched fractions of uninduced and 4 days induced TbPam16 RNAi cells were analyzed by SILAC-based quantitative mass spectrometry (same data set as in Fig 1B). (A–F) The indicated subgroups of mitochondrial proteins are highlighted in the indicated colors. The number of all more than 1.5-fold depleted proteins and the total number of all detected proteins for each subgroup are indicated in parentheses at the top of each panel. Numerical data for panel (A) and (F) are available in S1 Data. (TIF) [file pbio.3002449.s003.tif]

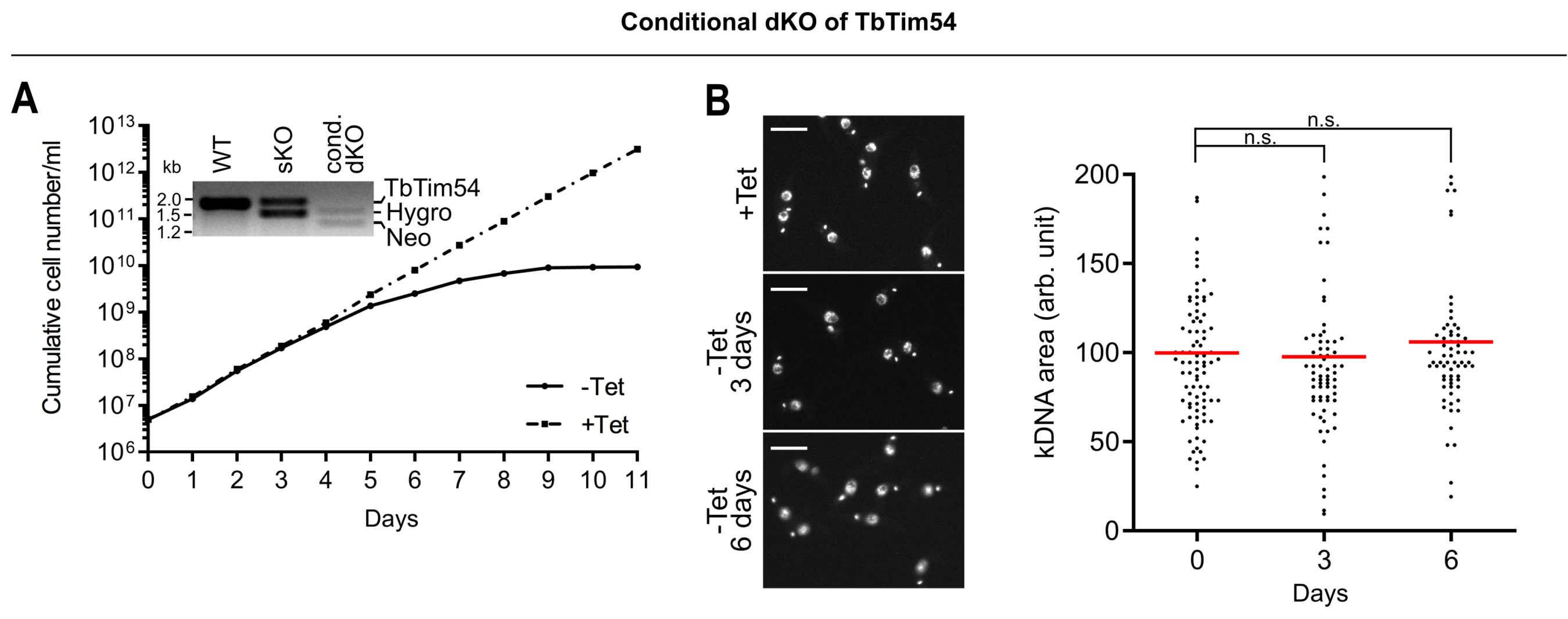

Supplement: S4 Fig — (A) Growth curve of a TbTim54 conditional double knockout (cond. dKO) cell line. While both TbTim54 alleles are knocked out in uninduced (-Tet) as well as induced (+Tet) cells, TbTim54-HA is only ectopically expressed when tetracycline is present. Inset: Verification of single knockout (sKO) and dKO by PCR using a primer pair to amplify the TbTim54 ORF (approximately 2.0 kilobases (kb)), the hygromycin (hygro, approximately 1.5 kb), or the neomycin (neo, approximately 1.3 kb) resistance cassettes at the same time. Hygro was used to replace the first and neo was used to replace the second allele. (B) Left: DAPI-stained TbTim54 cond. dKO cell line grown in the presence of tetracycline (+Tet) and 3 or 6 days after the removal of tetracycline from the medium (-Tet). Right: Quantification of kDNA areas in 69 to 90 DAPI-stained cells. The red line indicates the mean of the kDNA areas for each time point. The mean of the control cells (+Tet) was set to 100%. n.s.: not significant (P value >0.05) as calculated by an unpaired two-tailed t test. Numerical data for panel (A) and (B) are available in S1 Data. (TIF) [file pbio.3002449.s004.tif]

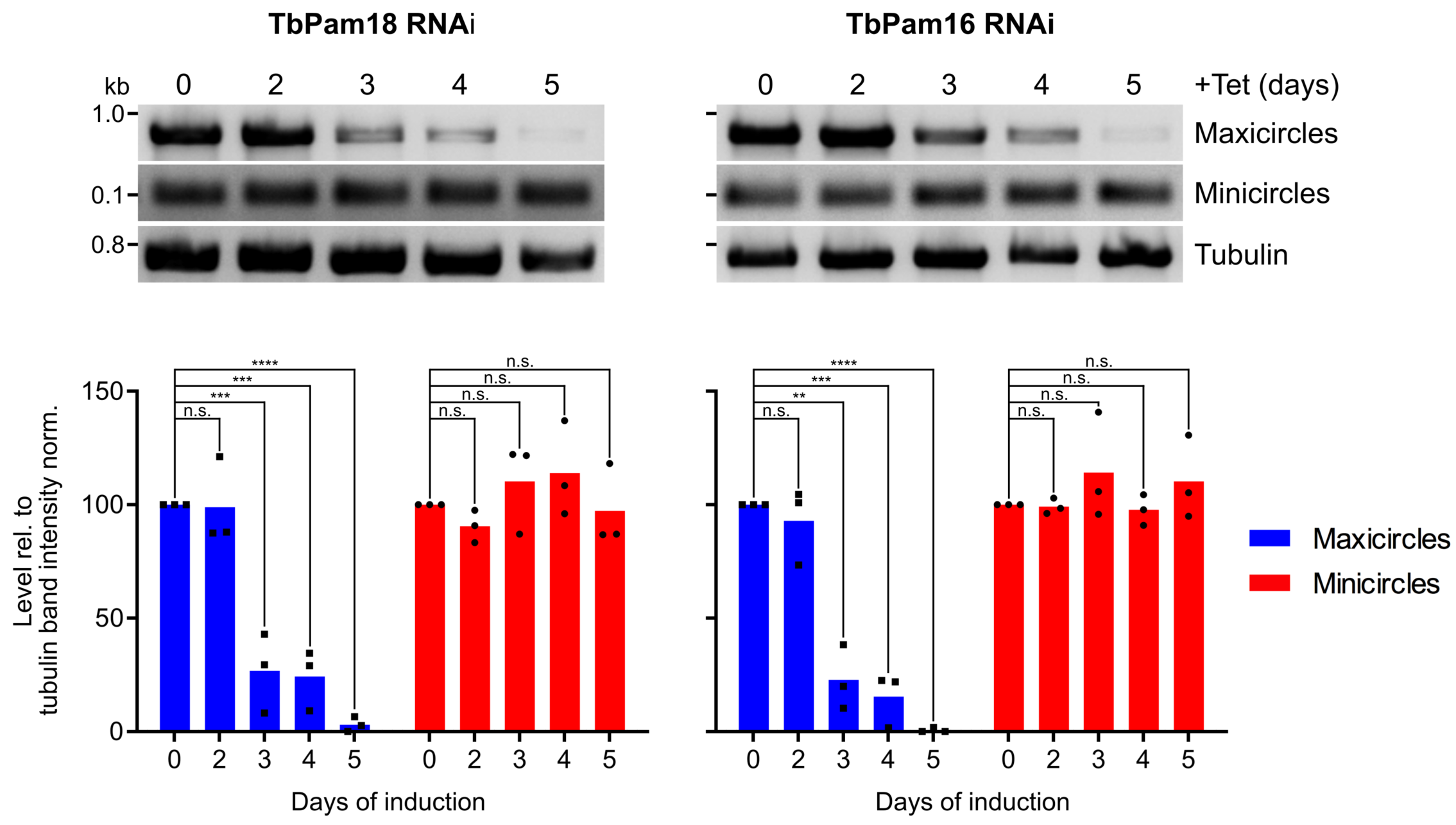

Supplement: S5 Fig — Upper panels: A quantitative PCR-based (qPCR) method was used to detect changes in steady-state levels of total mini- and maxicircles. Total DNA was extracted from uninduced and 2 to 5 days induced TbPam18 and TbPam16 RNAi cell lines. This DNA was used as the template in PCRs amplifying specific mini- and maxicircle regions or the intergenic region of tubulin. PCR products were analyzed on agarose gels. Lower panels: Densitometric quantification of mini- and maxicircle abundance as detected by qPCR. The ratio of the mini- or maxicircle band intensity and the respective control (tubulin band intensity) was normalized (norm.) to the ratios of uninduced cells. Blue (maxicircles) and red (minicircles) bars represent the mean of 3 independent biological replicates. n.s.: not significant, **: P value <0.05, ***: P value <0.005, ****: P value <0.0001, as calculated by an unpaired two-tailed t test. Numerical data are available in S1 Data. (TIF) [file pbio.3002449.s005.tif]

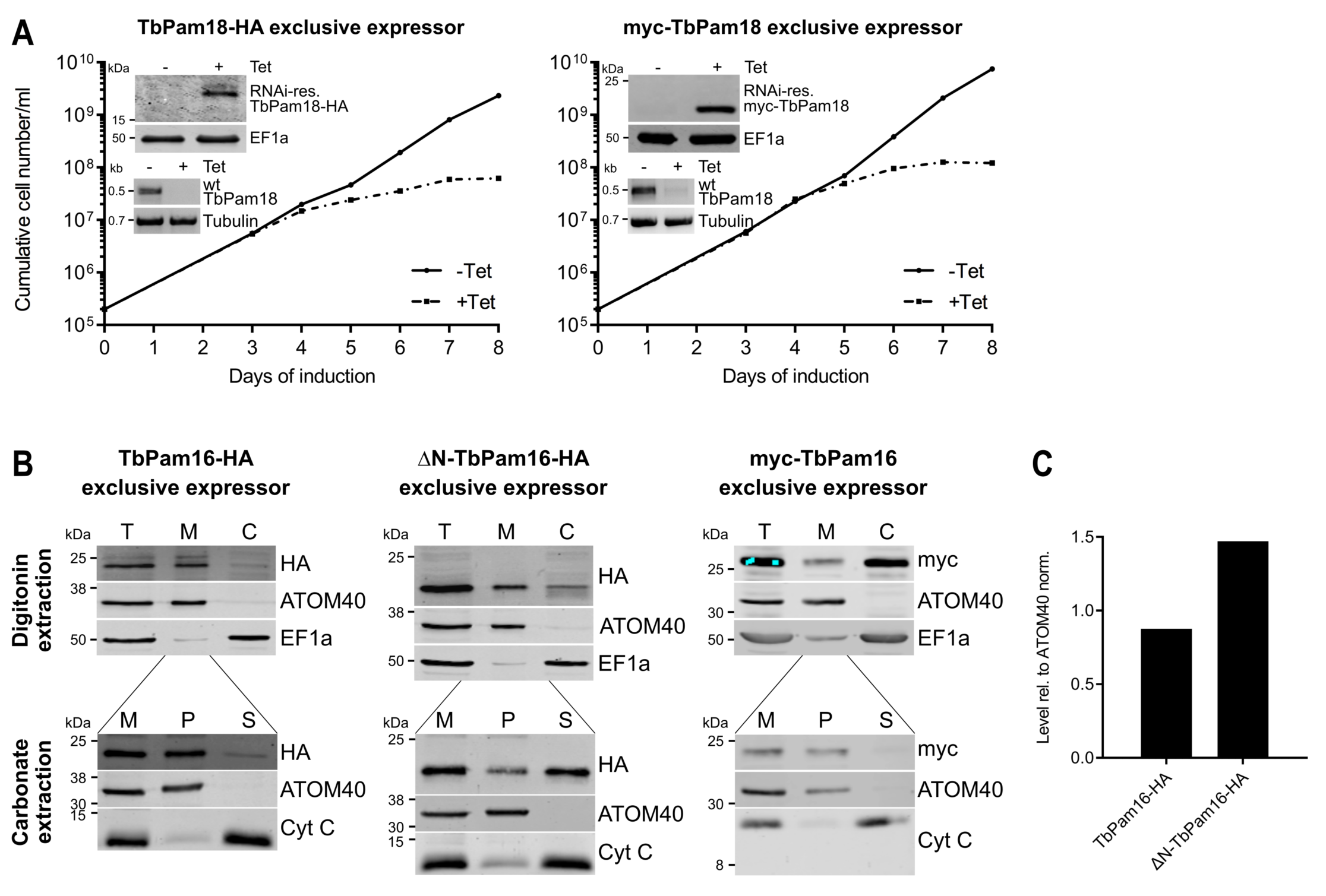

Supplement: S6 Fig — (A) Growth curve of uninduced (-Tet) and induced (+Tet) cells expressing RNAi-resistant (RNAi-res.) TbPam18-HA (left) or myc-TbPam18 (right) in the background of RNAi against the wild type (wt) TbPam18 (TbPam18-HA and myc-TbPam18 exclusive expressors). Insets, top: Immunoblot analysis of whole cell extracts of uninduced (-) and 2 days induced (+) cells, probed for RNAi-res. TbPam18-HA or myc-TbPam18 and EF1a as loading control. Insets, bottom: RT-PCR products of the wt TbPam18 mRNA in uninduced (-) or 2 days induced (+) cells. Tubulin mRNA serves as loading control. (B) Upper panels: Immunoblot analysis of total cells (T), digitonin-extracted, mitochondria-enriched (M), and soluble cytosolic (C) fractions of TbPam16-HA, ΔN-TbPam16-HA, and myc-TbPam16 exclusive expressor cell lines. Blots were probed with anti-HA antibodies and antisera against ATOM40 and EF1a, which serve as mitochondrial and cytosolic markers, respectively. Lower panels: digitonin-extracted, crude mitochondrial fractions (M) were subjected to an alkaline carbonate extraction resulting in a pellet enriched in integral membrane proteins (P) and a soluble supernatant fraction (S). Immunoblots were probed with anti-HA and antisera against ATOM40 and cytochrome C (Cyt C), which serve as marker for integral membrane and soluble proteins, respectively. All immunoblots derive from the same gel. (C) Quantification of the expression levels in total cells, lanes T in (B), of TbPam16-HA and ΔN-TbPam16-HA normalized to ATOM40. Numerical data for panel (A) and (C) are available in S1 Data. (TIF) [file pbio.3002449.s006.tif]

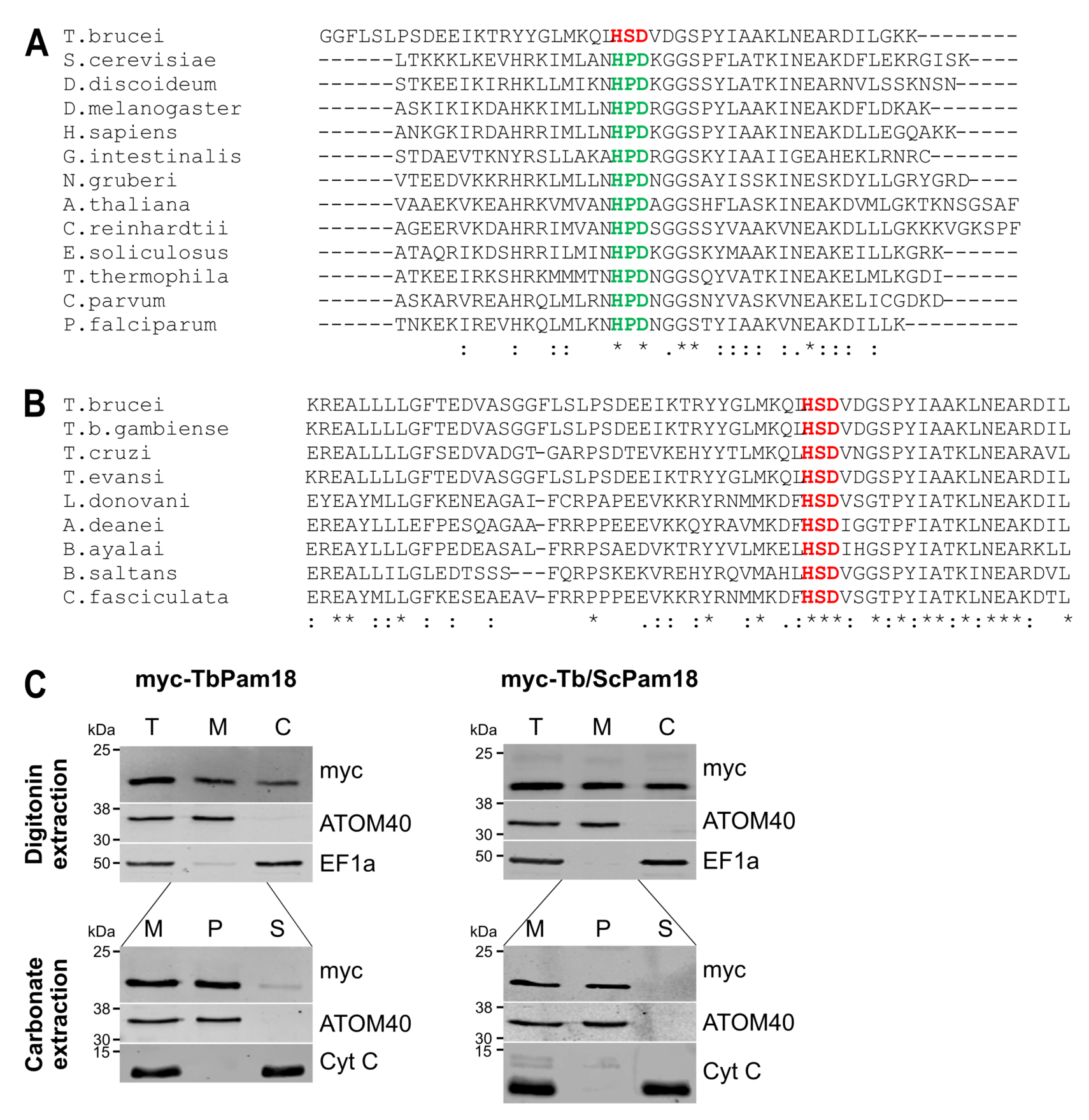

Supplement: S7 Fig — (A) Sequence alignment of N-terminal regions of Pam18 homologues of 13 representative eukaryotes. (B) Sequence alignment of N-terminal regions of Pam18 homologues of 9 representative trypanosomatids. In (A) and (B) Histidine-Proline-Aspartate (HPD) motifs are highlighted in red and Histidine-Serine-Aspartate (HSD) motifs in green. (C) Upper panels: Immunoblot analysis of total cells (T), digitonin-extracted mitochondria-enriched (M), and soluble cytosolic (C) fractions of cell lines expressing N-terminally myc-tagged TbPam18 or Tb/ScTbPam18. Blots were probed with anti-myc antibodies and antisera against ATOM40 and EF1a, which serve as mitochondrial and cytosolic markers, respectively. Lower panels: digitonin-extracted crude mitochondrial fractions (M) were subjected to an alkaline carbonate extraction resulting in a pellet enriched in integral membrane proteins (P) and a soluble supernatant fraction (S). Immunoblots were probed with anti-myc and antisera against ATOM40 and Cyt C, which serve as makers for integral membrane and soluble proteins, respectively. (TIF) [file pbio.3002449.s007.tif]

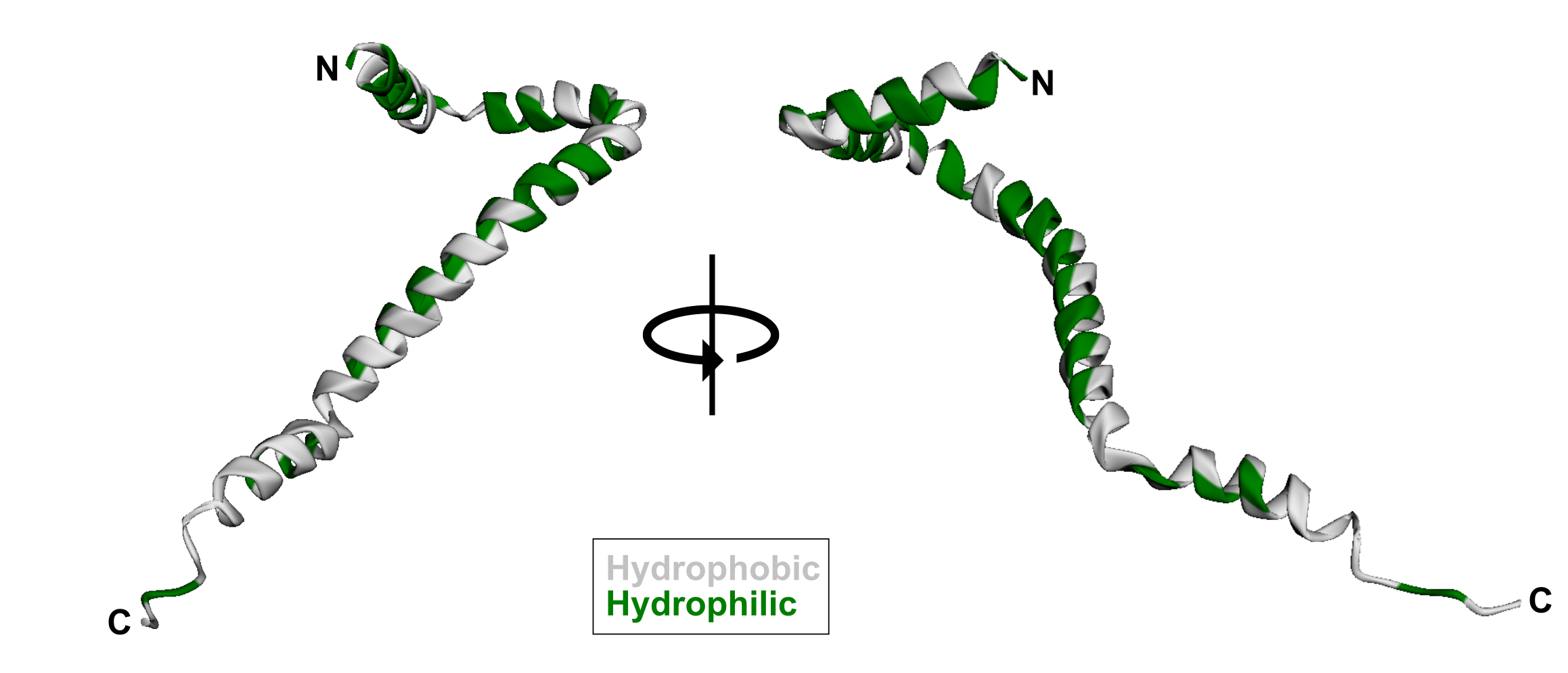

Supplement: S8 Fig — (TIF) [file pbio.3002449.s008.tif]

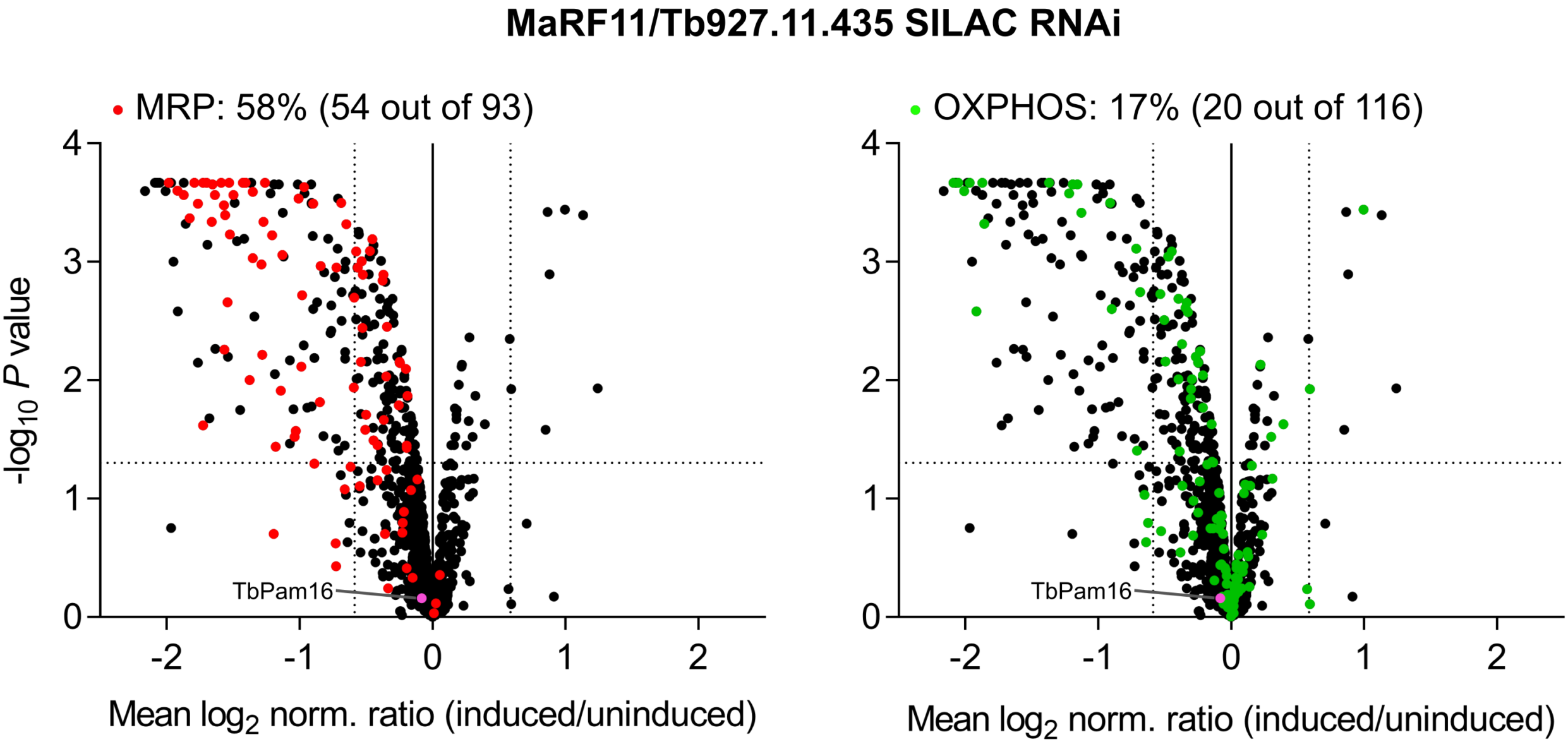

Supplement: S9 Fig — Mitochondria-enriched fractions of uninduced and 4 days induced MaRF11 RNAi cells were analyzed by SILAC-based quantitative mass spectrometry. The data set was filtered for mitochondrial proteins and the mean log2 of normalized ratios (induced/uninduced) was plotted against the corresponding negative log10 of adjusted P value (limma test). Highlighted are mitochondrial ribosomal proteins (MRPs, red) and components of the oxidative phosphorylation pathway (OXPHOS, green) as well as TbPam16 (pink). The horizontal dotted line in each volcano plot marks an adjusted P value of 0.05. The vertical dotted lines indicate a fold-change in protein abundance of ±1.5. The percentages of all detected MRPs or OXPHOS proteins that are depleted more than 1.5-fold is indicated at the top of each panel. The number of all more than 1.5-fold depleted MRPs or OXPHOS proteins and the total number of all detected MRPs or OXPHOS proteins is shown in parentheses. MaRF11/Tb927.11.435 was not detected. Numerical data are available in S3 Table. (TIF) [file pbio.3002449.s009.tif]

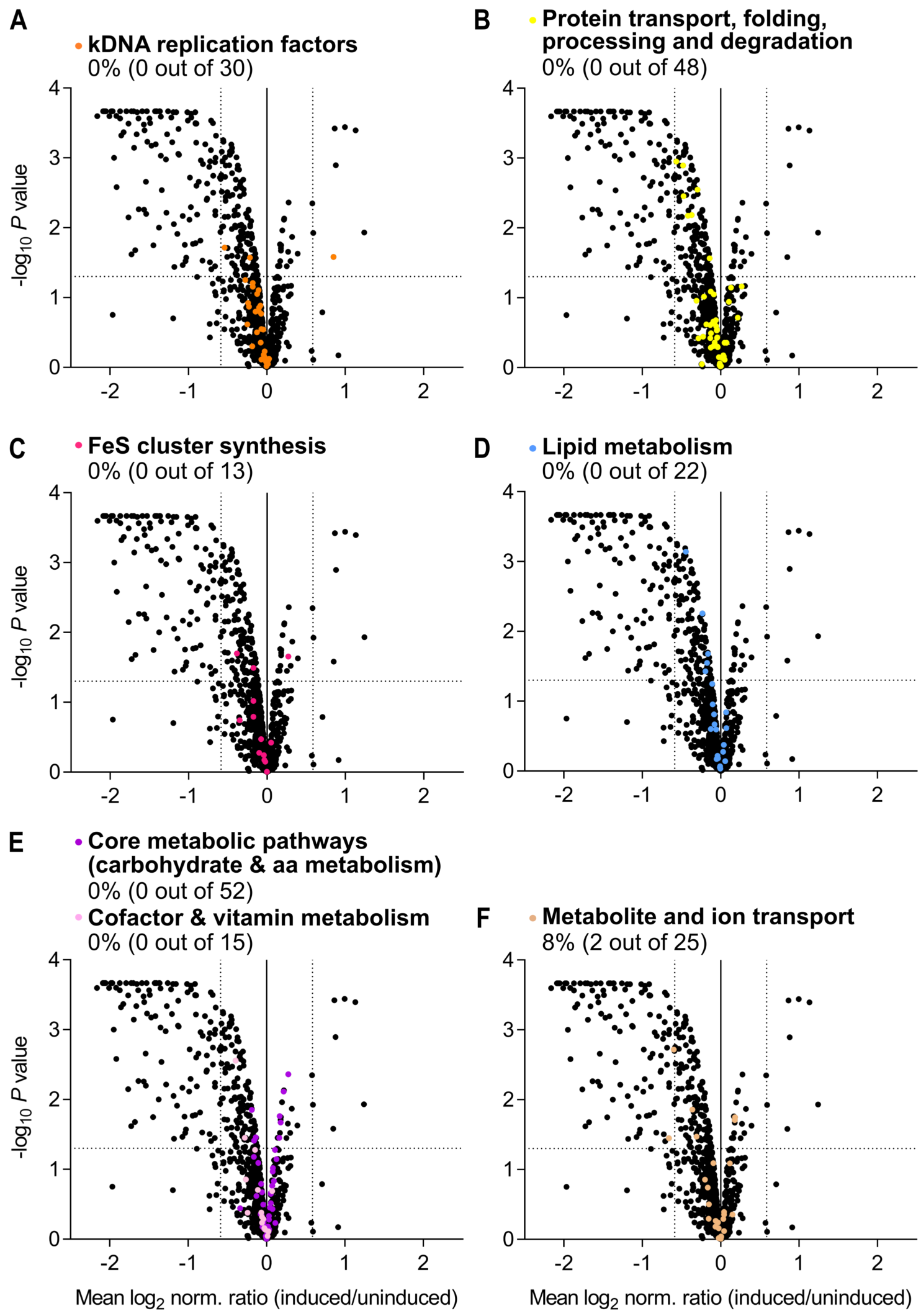

Supplement: S10 Fig — Mitochondria-enriched fractions of uninduced and 4 days induced MaRF11 RNAi cells were analyzed by SILAC-based quantitative mass spectrometry (same data set as in S9 Fig). (A–F) The indicated subgroups of mitochondrial proteins are highlighted in the indicated colors. The number of all more than 1.5-fold depleted proteins and the total number of all detected proteins for each subgroup are indicated in parentheses at the top of each panel. Numerical data for panel are available in S3 Table. (TIF) [file pbio.3002449.s010.tif]

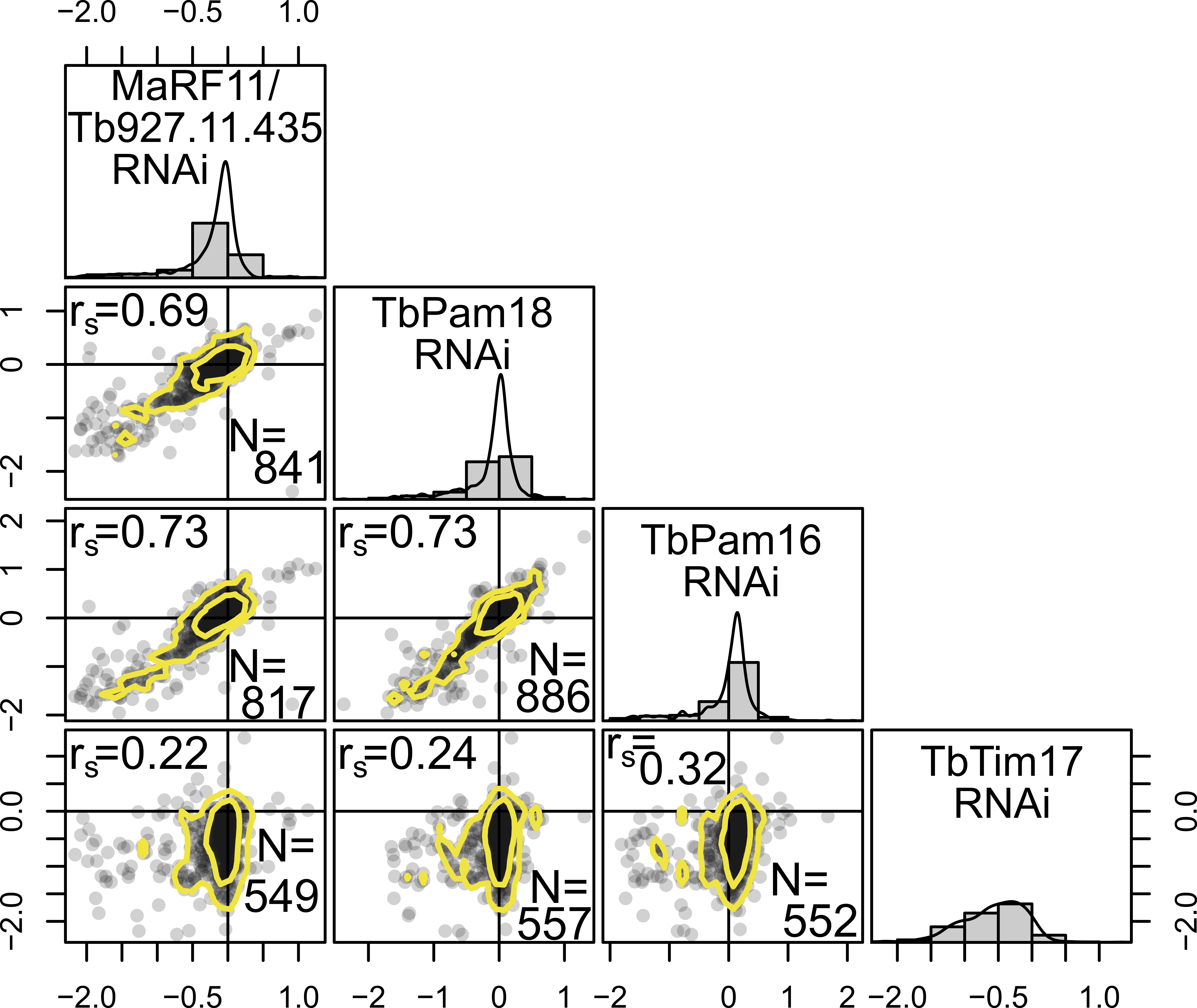

Supplement: S11 Fig — The mitochondrial proteins detected in the data sets of the indicated SILAC RNAi analyses were quantitatively compared in a pairs diagram created with R (version 4.2.1). The log2-normalized foldchanges of all detected mitochondrial proteins of 1 SILAC experiment were compared to each of the other experiments in individual scatter graphs. Yellow clouds indicate the range in which 50% (inner cloud) and 90% (outer cloud) of the data points are located. The correlation between the data sets was calculated using the Spearman’s rank correlation algorithm and is indicated as rs. N indicates the total number of proteins that could be compared between 2 data sets. The 4 panels along the diagonal axis contain histograms combined with density graphs displaying the overall data point distribution in each experiment. As all 4 data sets have a left-skewed distribution, the Spearman’s rank correlation was used as the statistical measure. Numerical data are available in S1 and S3 Tables. (TIF) [file pbio.3002449.s011.tif]

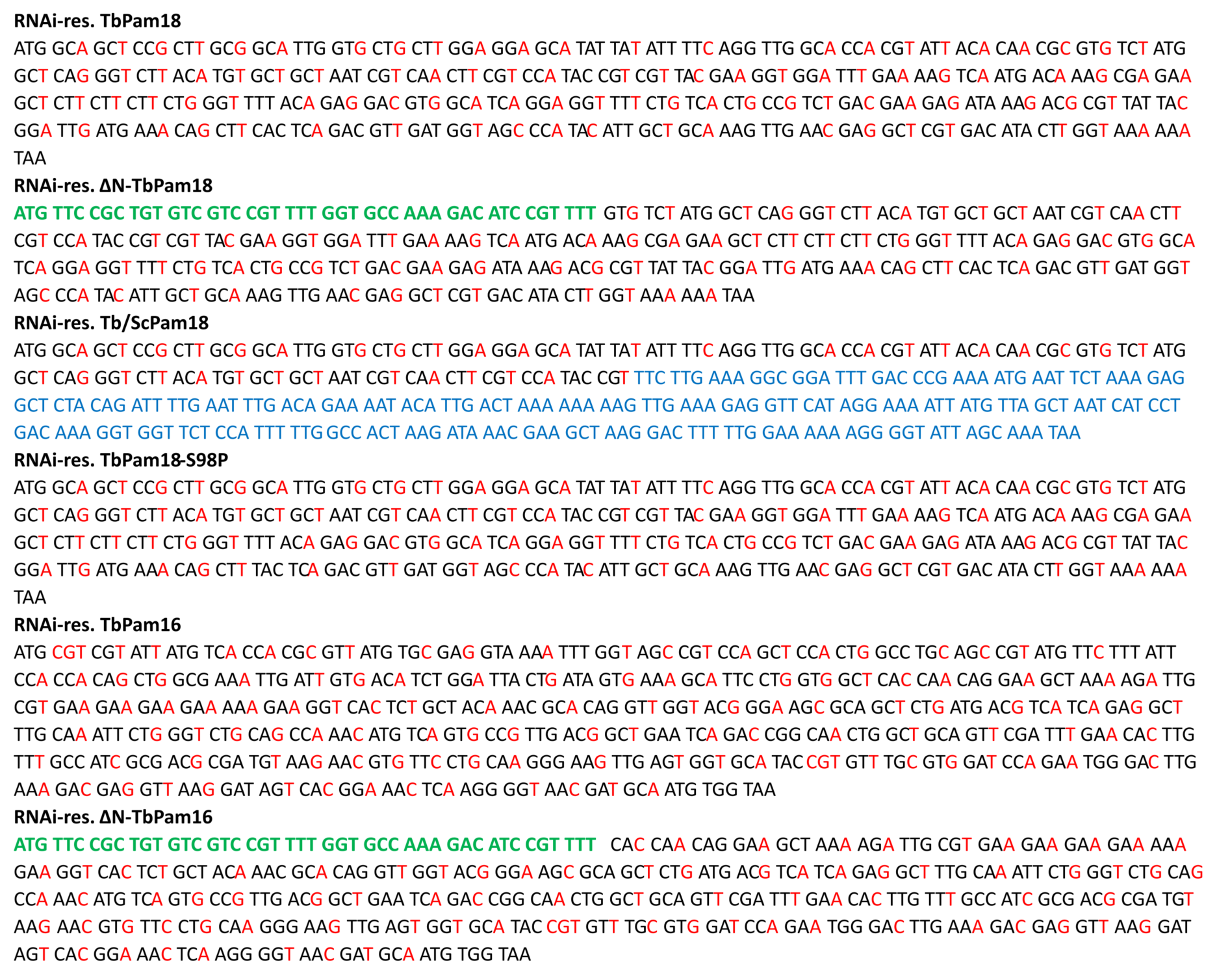

Supplement: S12 Fig — RNAi-resistant (RNAi-res.) TbPam18 and TbPam16 DNA sequences in black with changed nucleotides highlighted in red. In the ΔN-TbPam18 and ΔN-TbPam16 constructs, the first 81 and 156 nucleotides, respectively, were replaced by the first 45 nucleotides of TbmHsp60, which encode the mitochondrial targeting sequence of the protein (green). To generate the Tb/ScPam18 fusion protein, the first 295 nucleotides of RNAi-res. TbPam18 were fused to the last 213 nucleotides of yeast (Sc) Pam18 (blue). To generate RNAi-res. TbPam18-S98P, the cytosine at position 292 of the nucleotide sequence was exchanged against a thymine (highlighted in yellow). (TIF) [file pbio.3002449.s012.tif]
