# Supplementary material for: Pam16 and Pam18 were repurposed during Trypanosoma brucei evolution to regulate the replication of mitochondrial DNA
Source: PLoS Biol. 2024 Aug 15;22(8):e3002449. doi: 10.1371/journal.pbio.3002449 (PMC11349236; doi:10.1371/journal.pbio.3002449)

Raw images

Figure 1A (western blots)

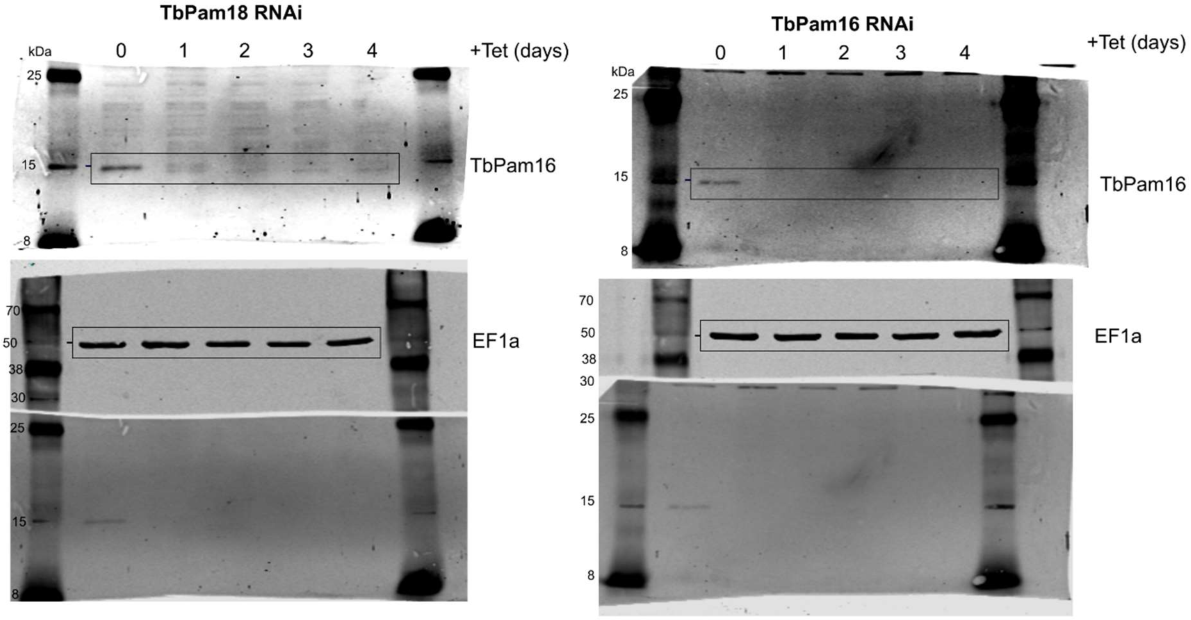

Figure 2B (Southern blots)

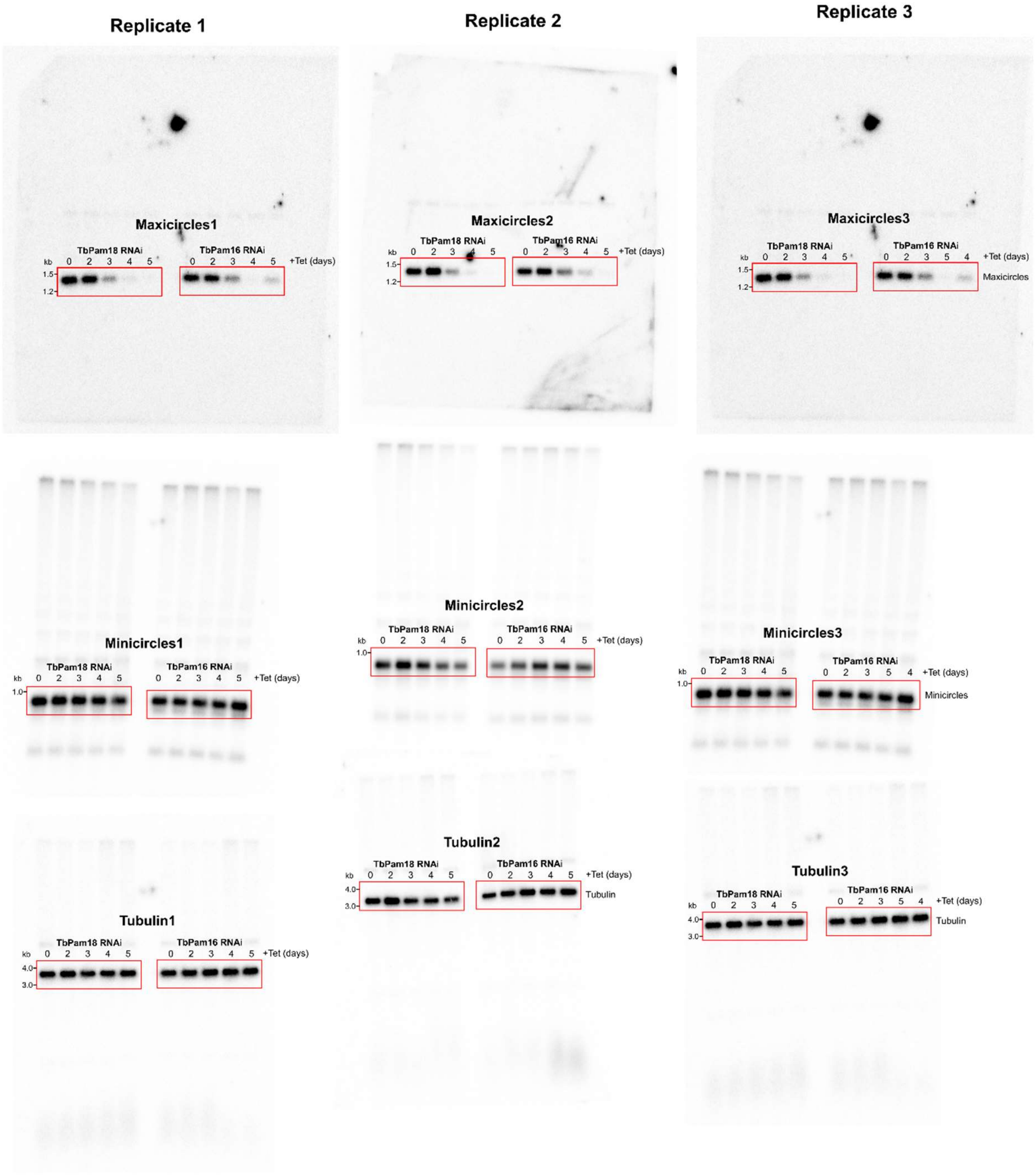

Figure 3A (EtBr-stained agarose gels)

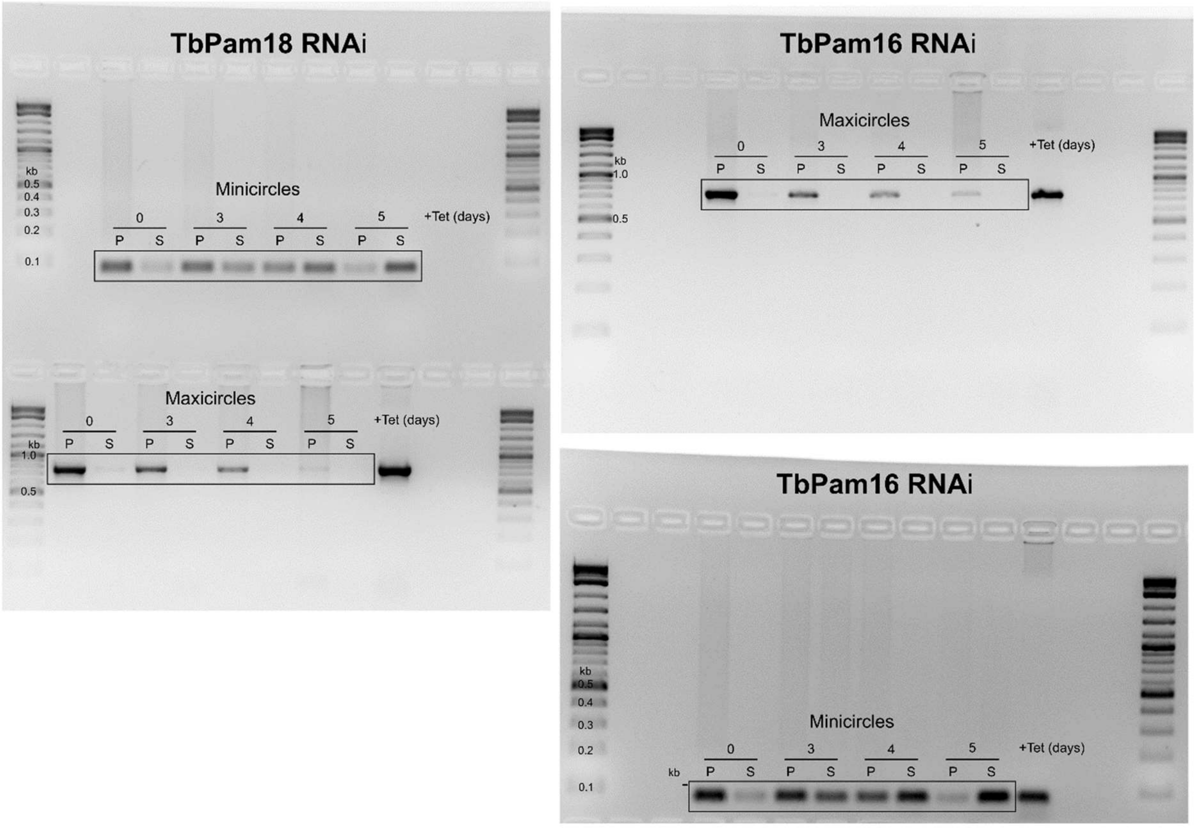

Figure 4A-B (EtBr-stained agarose gels)

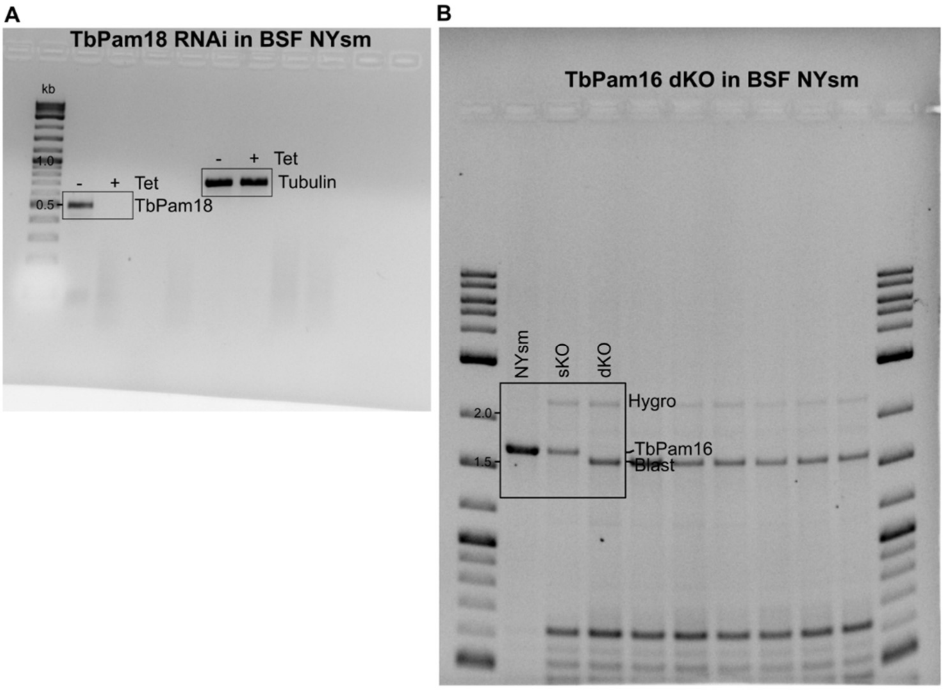

Figure 4D (Southern blots)

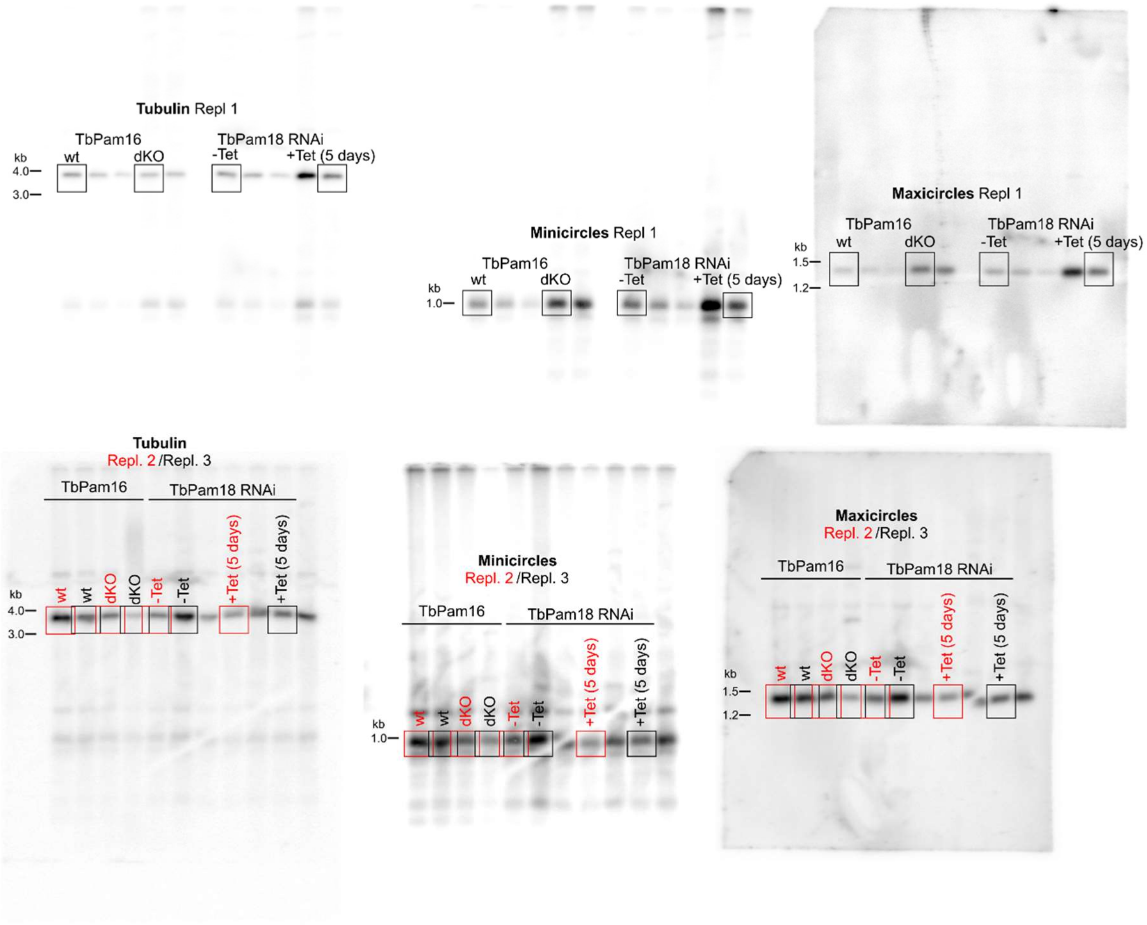

Figure 5B (northern blots and EtBr-stained agarose gels)

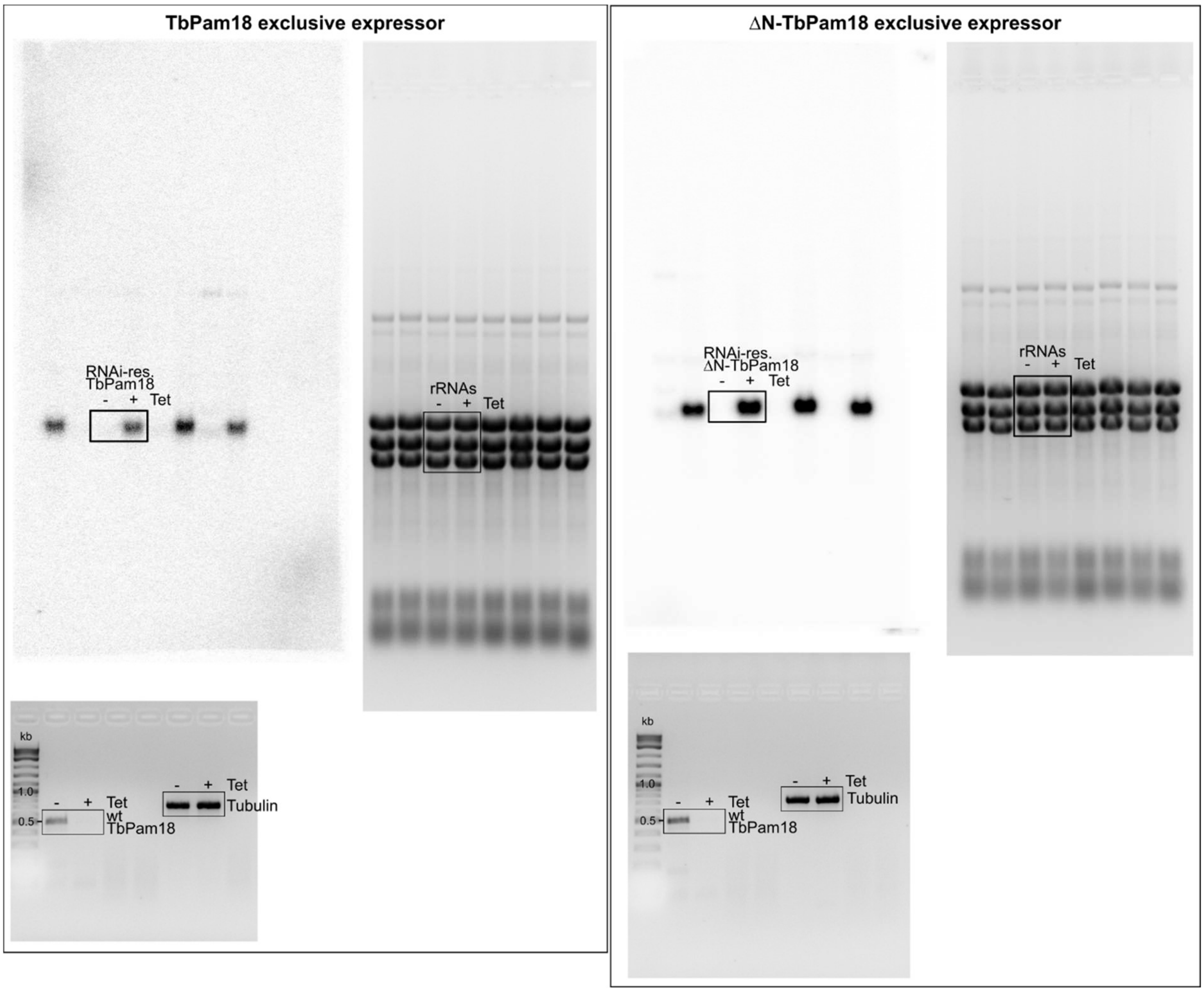

Figure 5C (western blots)

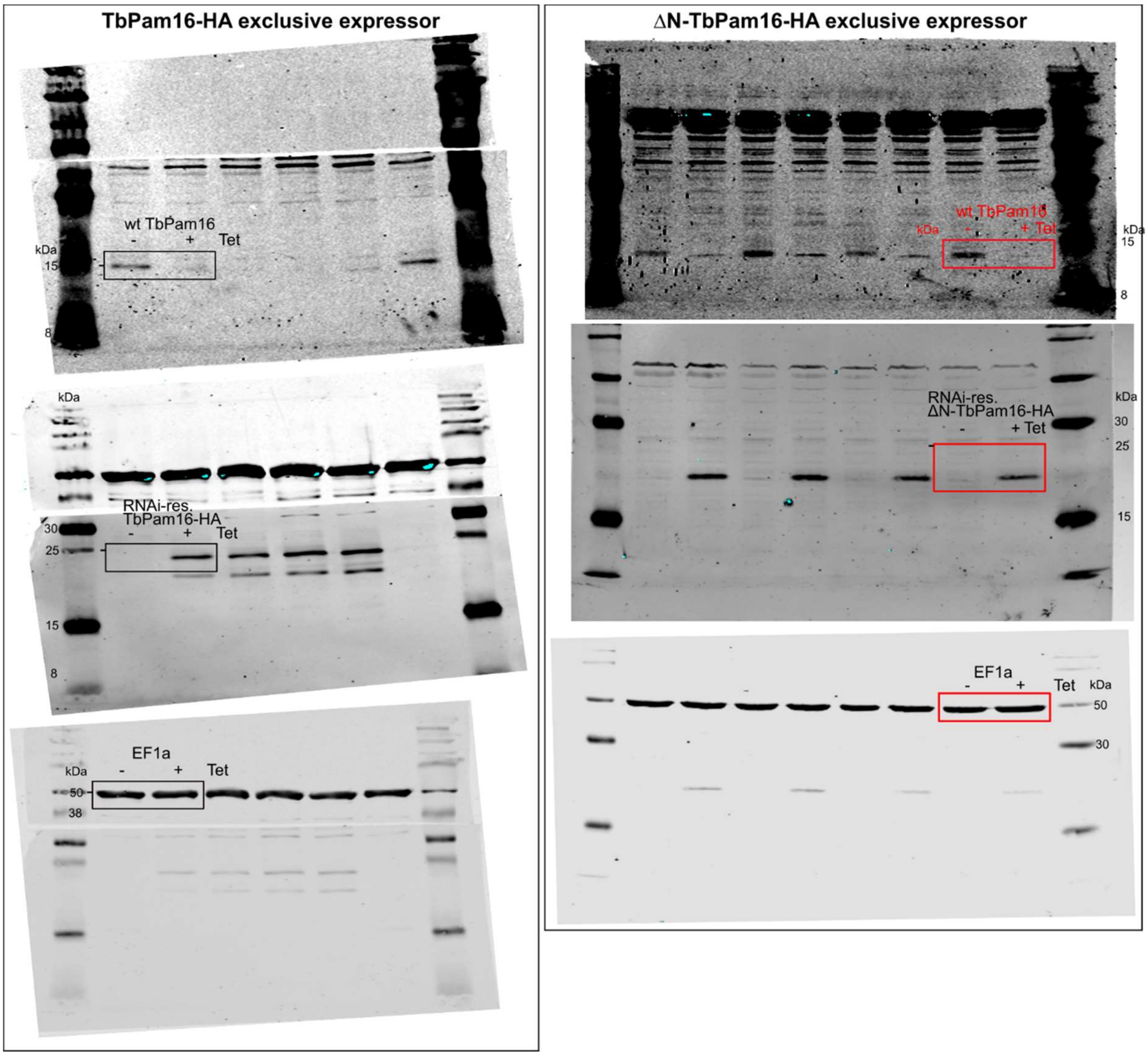

Figure 5D (left: western blot, middle: northern blot, right: EtBr-stained agarose gel)

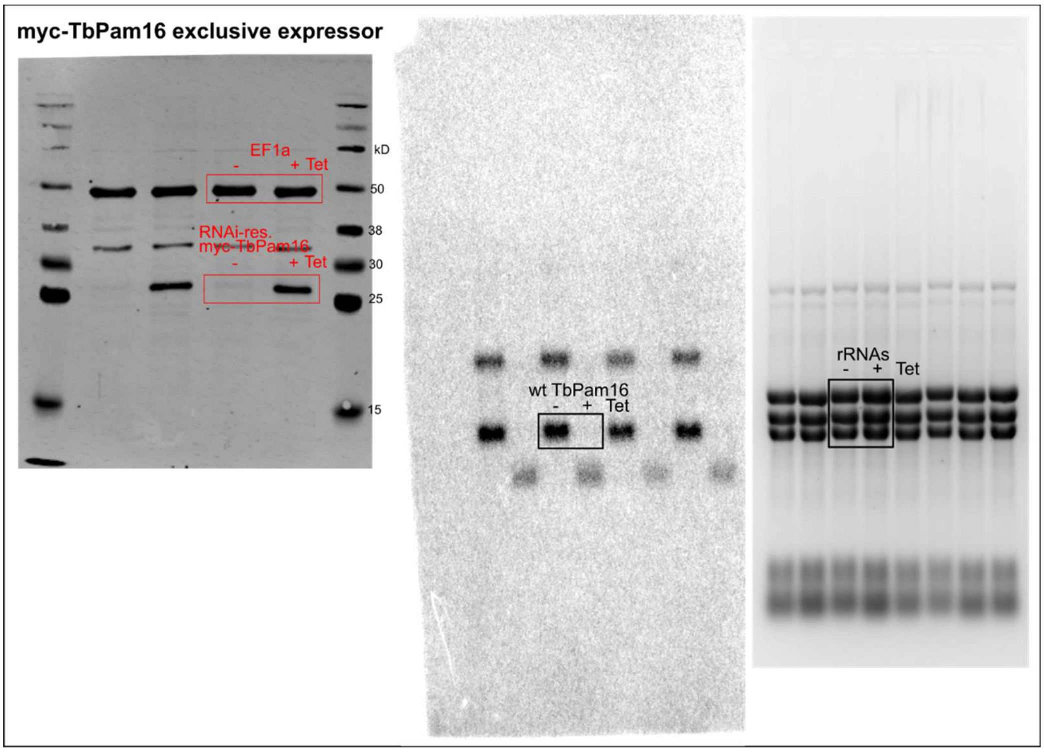

Figure 6B-C (B: EtBr-stained agarose gels, C: Northern blot and EtBr-stained agarose gels)

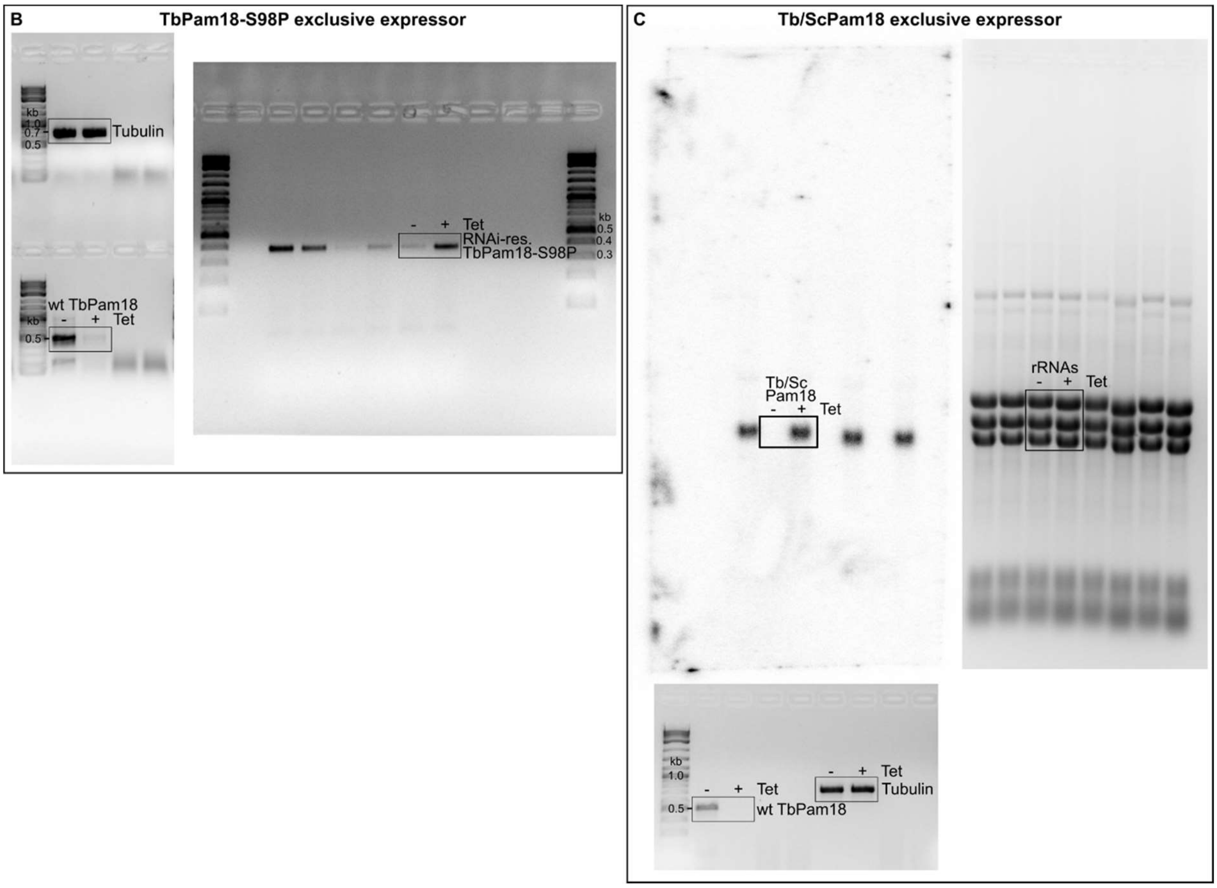

Figure 7B (northern blots and EtBr-stained agarose gels)

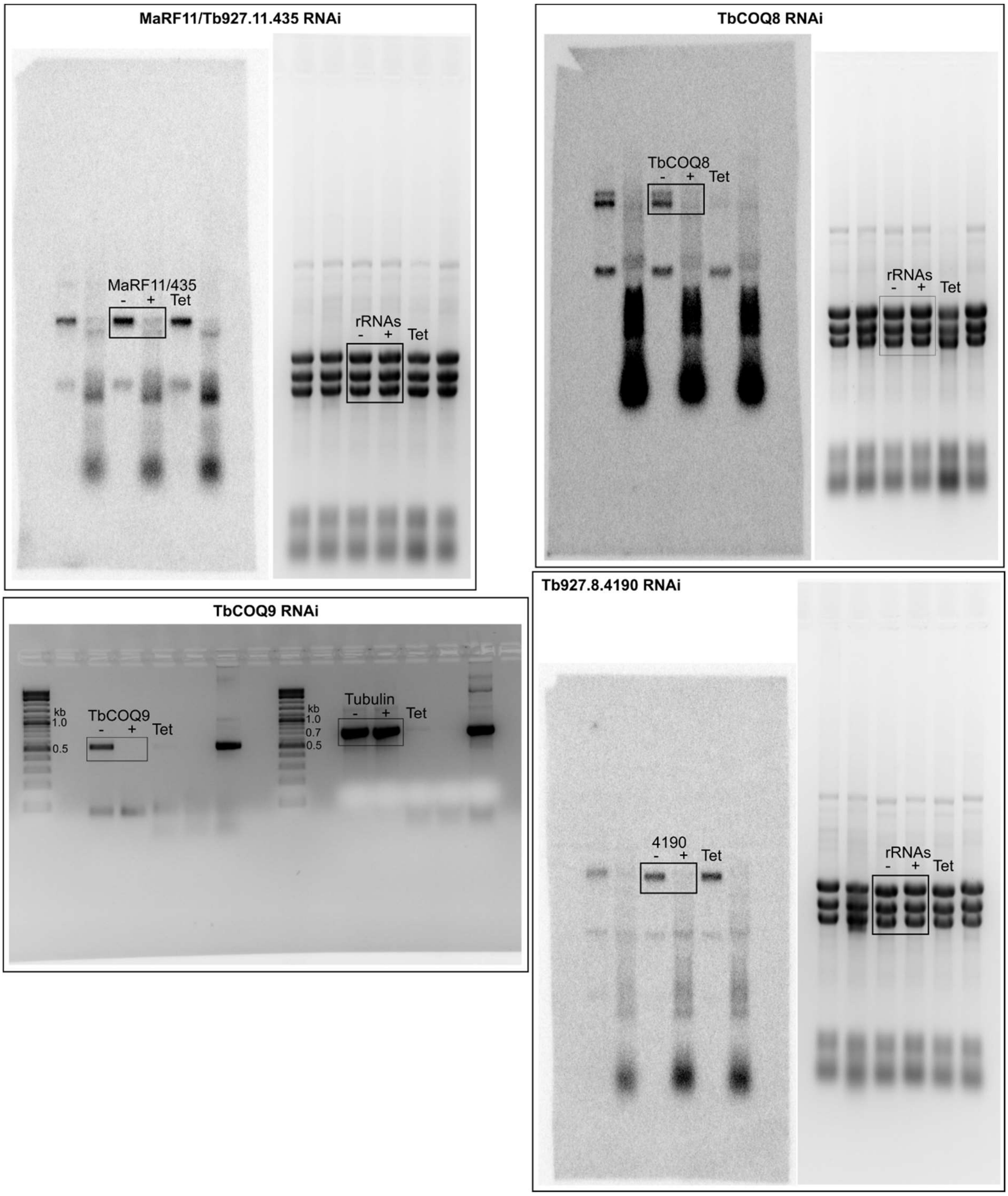

Figure 8B (EtBr-stained agarose gels)

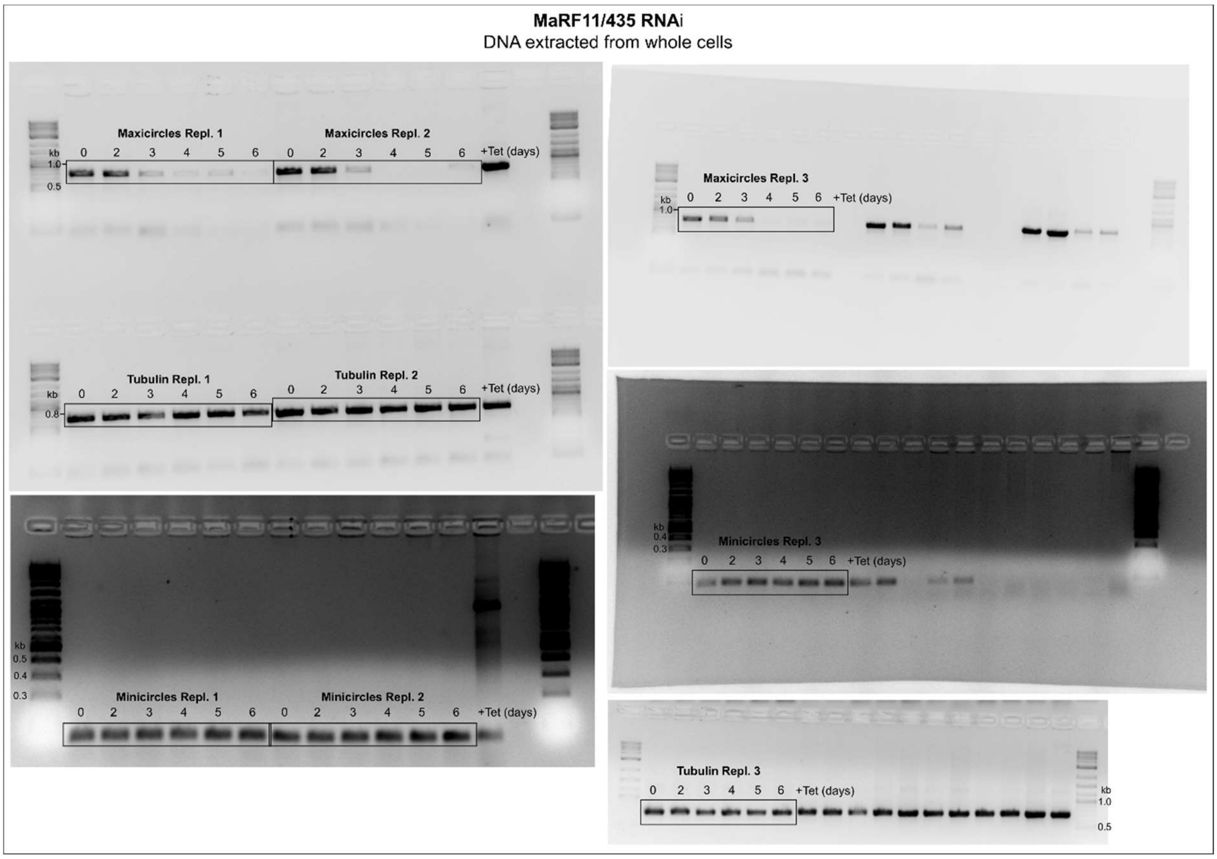

Figure 8C (EtBr-stained agarose gels)

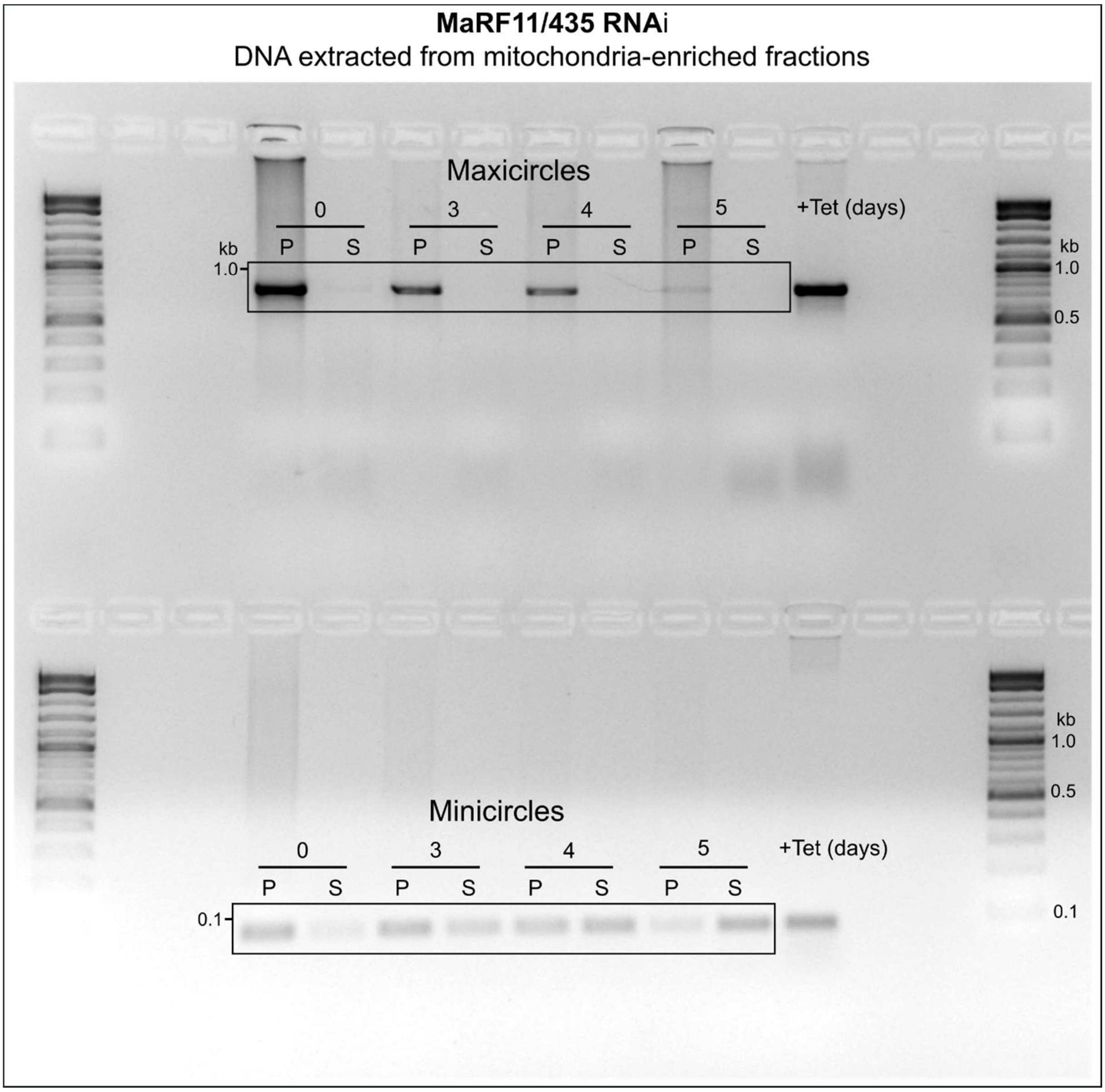

Figure 8D (EtBr-stained agarose gel)

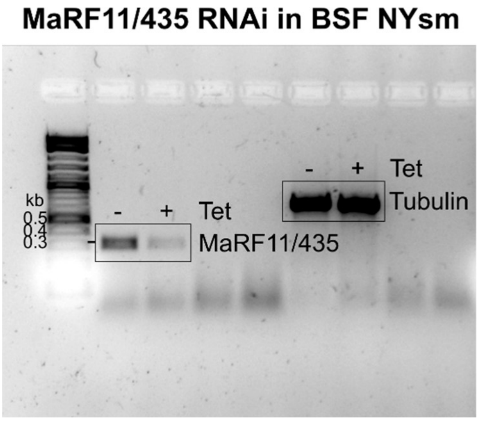

Figure 9A&C (A: EtBr-stained agarose gels, C: western blot)

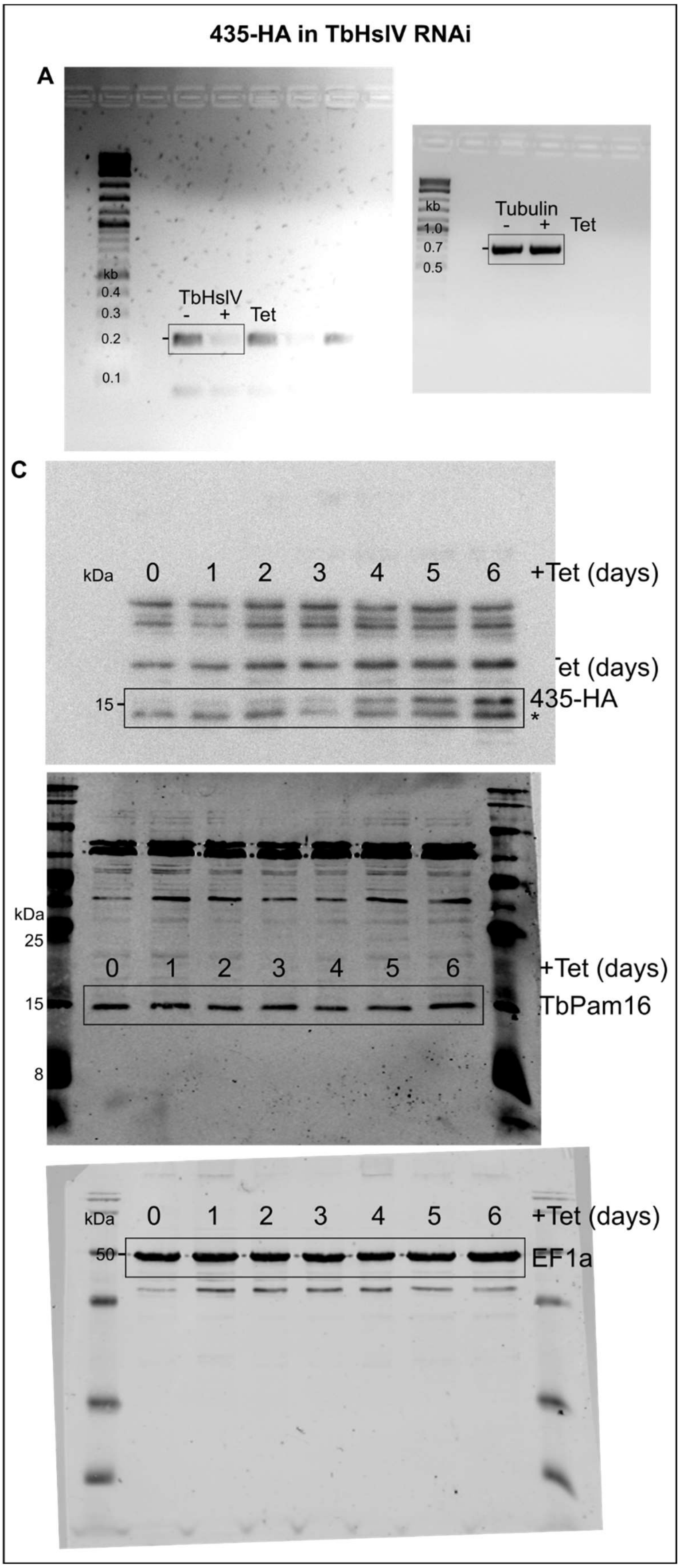

Figure S4A (EtBr-stained agarose gel)

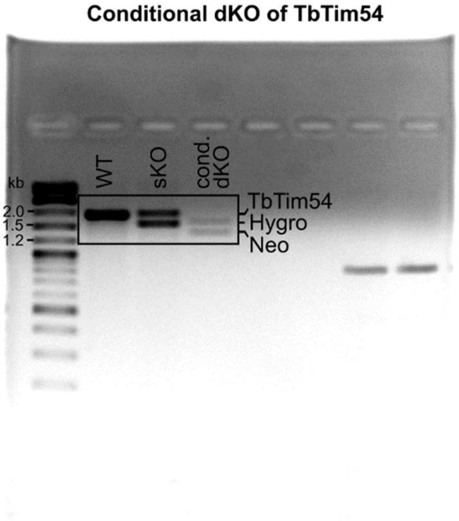

Figure S5 (EtBr-stained agarose gels)

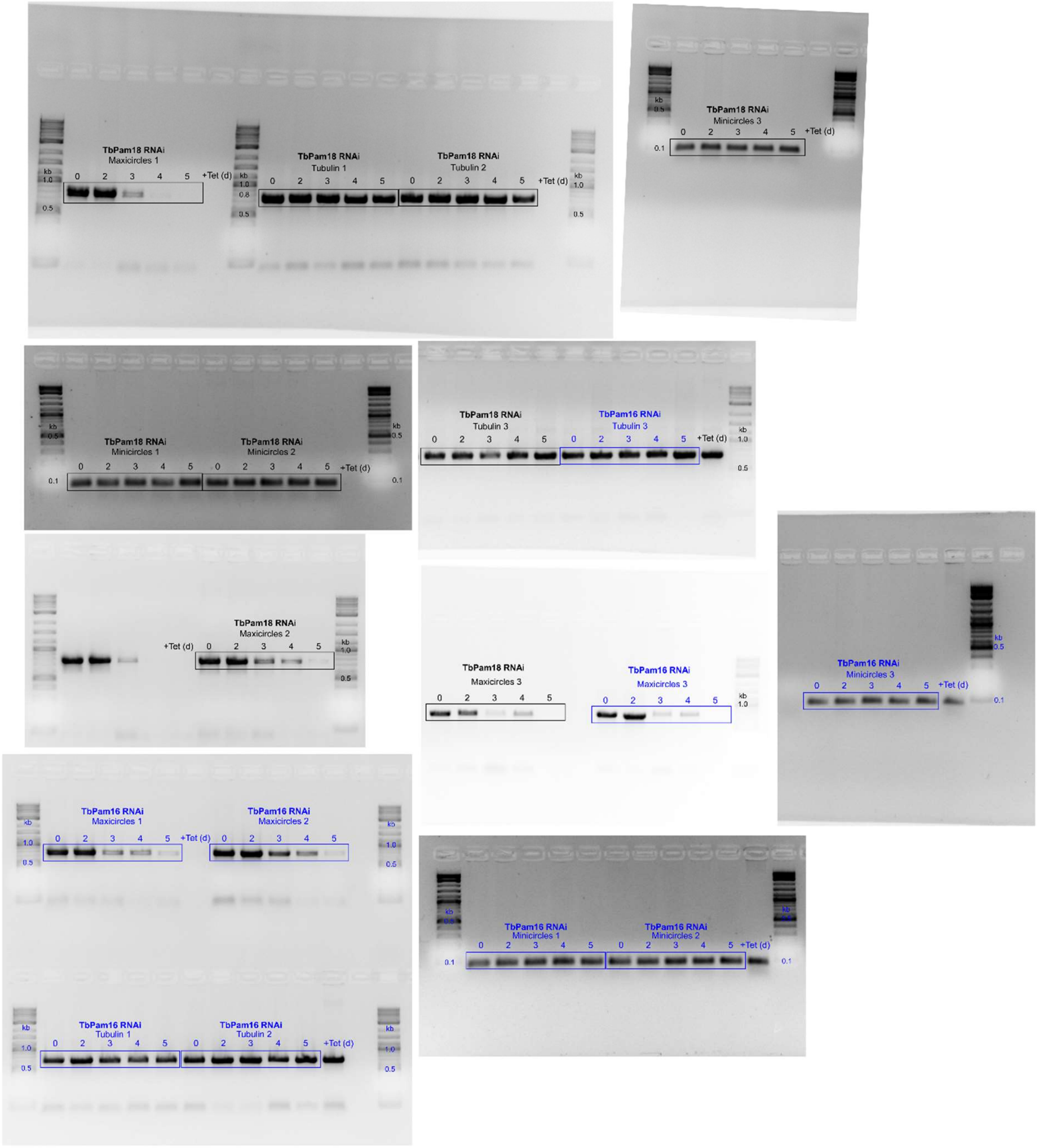

Figure 6A (western blots and EtBr-stained agarose gel)

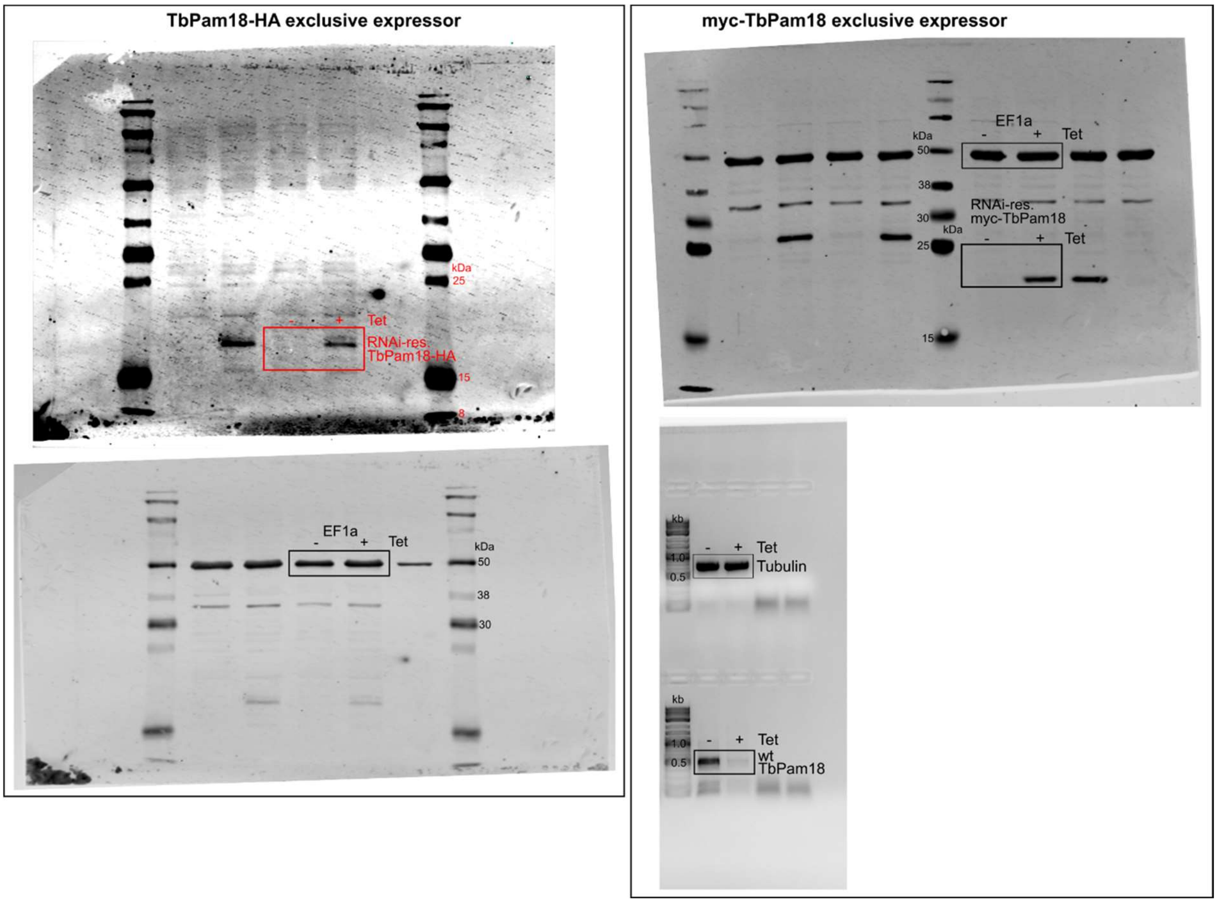

Figure 6B (western blots)

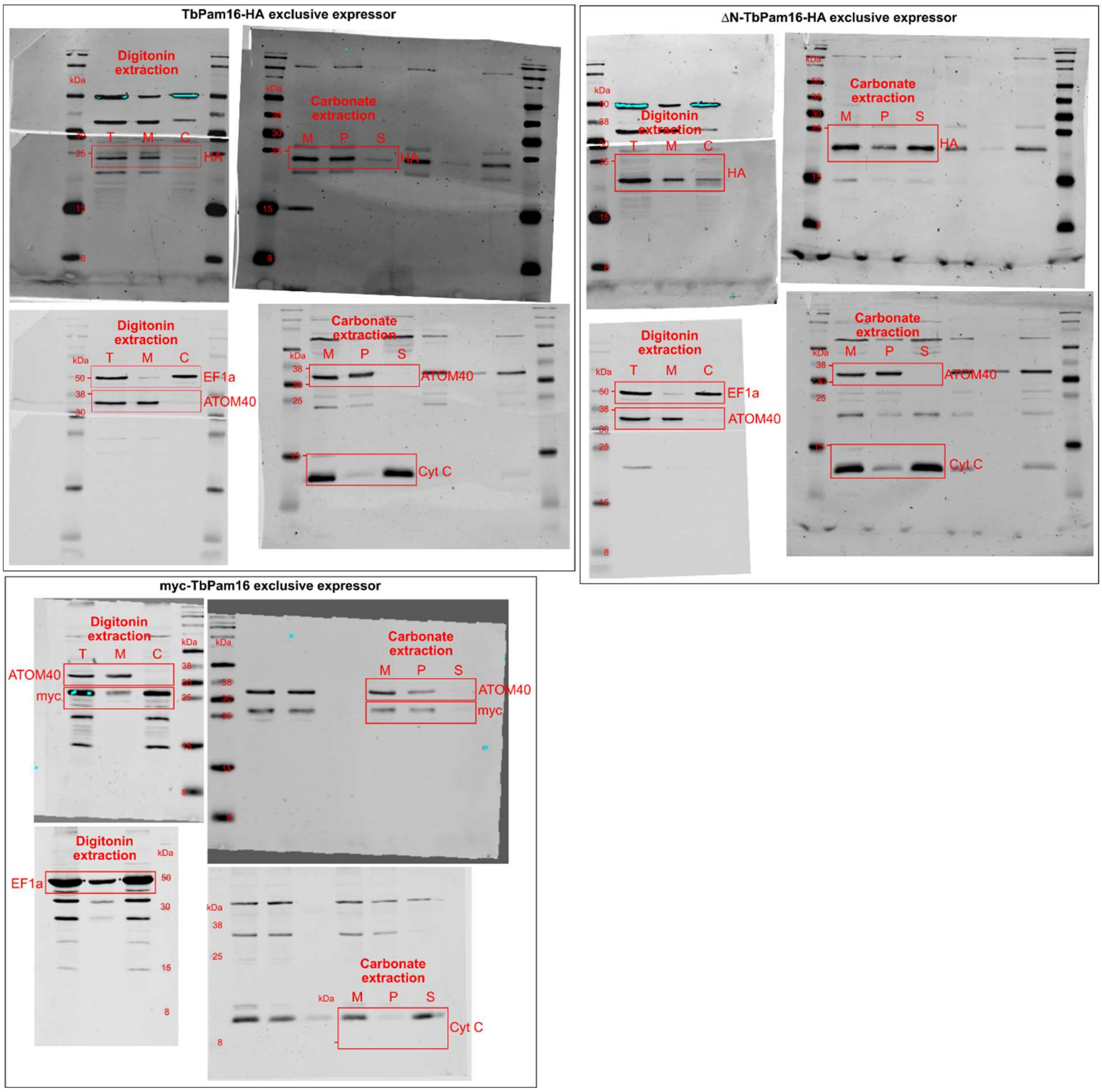

Figure S7C (western blots)

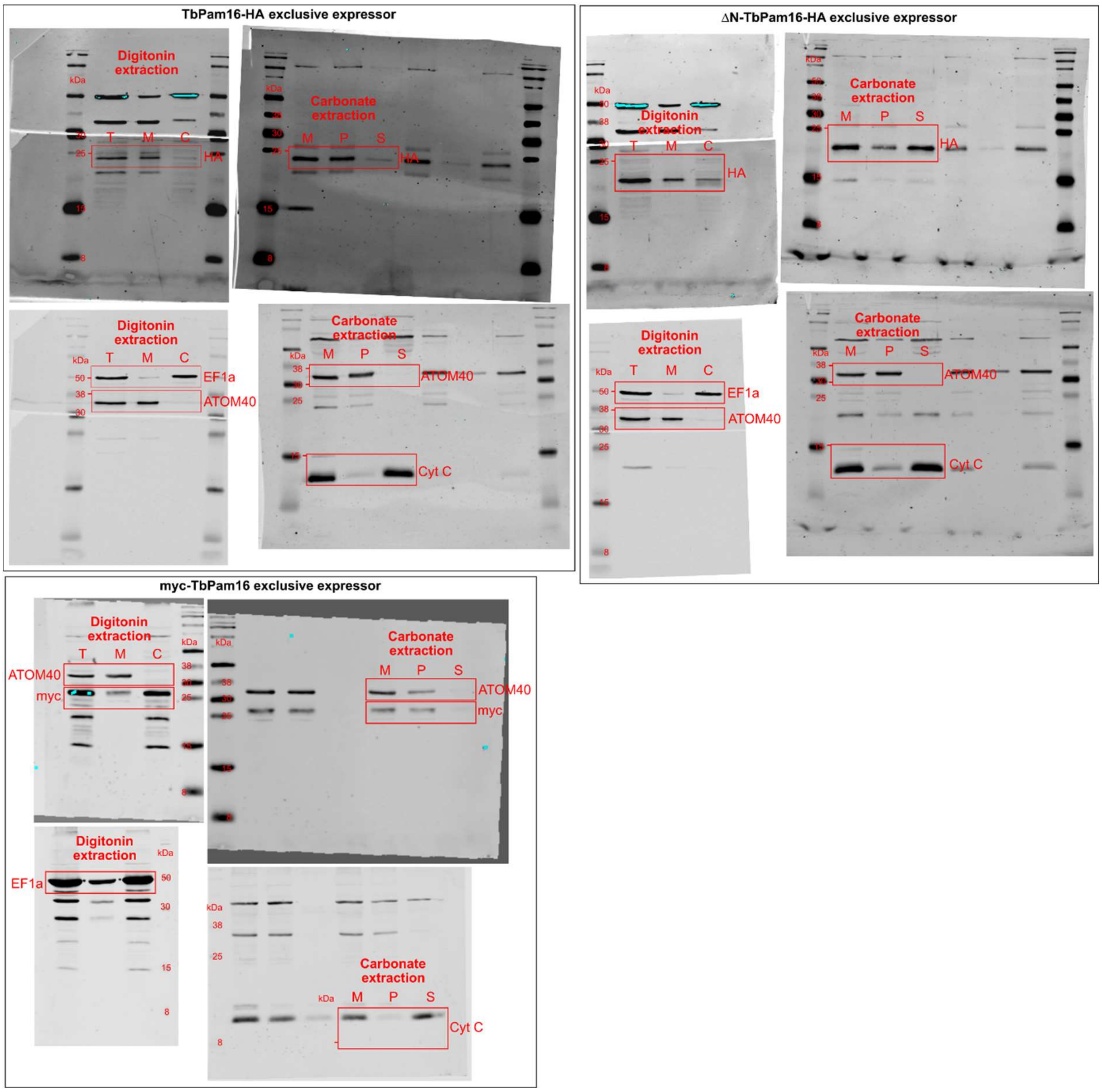

Supplement: S1 Raw Images — (PDF) [file pbio.3002449.s017.pdf]
