# Supplementary material for: Pam16 and Pam18 were repurposed during Trypanosoma brucei evolution to regulate the replication of mitochondrial DNA
Source: PLoS Biol. 2024 Aug 15;22(8):e3002449. doi: 10.1371/journal.pbio.3002449 (PMC11349236; doi:10.1371/journal.pbio.3002449)
Supplement: Attachment — Submitted filename: vonKaenel-Rebuttal-letter-1July.docx [file pbio.3002449.s018.docx]

**SUMMARY OF REVISIONS**

*All the reviewer’s comments have been addressed in the detail in the rebuttal letter below.*

*Here is a short summary:*

The three major questions the reviewers raised were:

1)Is it possible that TbPam18 and TbPam16 are involved in mitochondrial protein import and that the effect their depletion has on maxicircle replication is indirect?

*i) We provide new analyses and experiments which exclude that depletion of TbPam18 or TbPam16 affect protein import. SILAC RNAi analyses for both proteins show that mostly complexes containing mitochondrially encoded proteins get depleted, whereas protein categories containing only nuclear proteins are not affected (newly added Fig. S2 and S3).*

*ii) The TbPam18 and TbPam16 SILAC RNAi datasets (as well as the newly provided dataset for MaRF11, newly added Fig. S9, S10) were quantitatively compared with each other, and with the previously published TbTim17 SILAC RNAi dataset. TbTim17 is the core subunit of the single trypanosomal TIM complex. The results show a very high correlation between the TbPam18, TbPam16 and MaRF11 datasets but not with the one of TbTim17 (newly added Fig. S11).*

*iii) We show that inducible depletion of TbTim54 in a conditional double knock out cell line inhibits growth but does not affect kDNA size (newly added Fig. S4). TbTim54 is a putative protein import factor and one of the most downregulated proteins in the TbPam18 and TbPam16 depleted cells. This indicates that the kDNA loss seen in the TbPam18 and TbPam16 RNAi cell lines cannot be explained by co-depletion of TbTim54.*

2)Could it be that, even though a TbPam16 double knock cell line in bloodstream forms grows normally, maxicircles replication might still be reduced?

*We have now analyzed mini- and maxicircle abundance in bloodstream forms of the TbPam18 RNAi and the TbPam16 double knock out cell lines using DAPI-staining and Southern blots. Both cell lines grow normally (Fig. 4AB). We found no significant shrinkage of the kDNA disk in DAPI-stained cells after depletion of either of the two proteins (newly added Fig. 4C). Moreover, there was no significant change in the maxicircle and minicircle content when analyzed by Southern blots (newly added Fig. 4D).*

*As suggested by the editor we did not dig deeper into analyzing a putative non-essential function of the two proteins in bloodstream forms as the focus of our study is on the procyclic form.*

3)Is it possible to provide more evidence for the suggested model of how TbPam18, TbPam16 and MaRF11 may work?

*i) We have added the results of a new in vivo complementation experiment. It shows that in addition to the integral membrane localization also the very N-terminus of the IMS-exposed 33 aa domain appears to be essential for TbPam16 function (revised Fig. 5, S6). This is consistent with our speculation that the TbPam16/TbPam18/MaRF11 complex might be part of a circuit that transmits a signal from the nucleus via the IMS to the matrix to regulate maxicircle replication.*

*ii) We performed a SILAC RNAi analysis in a cell line depleted for MaRF11 (newly added Fig. S9, S10). The results show that depletion of membrane-bound TbPam18 and TbPam16, as well as the soluble protein MaRF11 cause exactly the same phenotypes, strongly suggesting that all three proteins are involved in the same biological process.*

*- However, we were not able to provide evidence for the direct involvement of MaRF11 in maxicircle replication, this is now explicitly stated in the manuscript. Moreover, we clearly emphasize the limitations of our model (last paragraph in the discussion section and revised Fig. 10)*

*iii) We report our attempts to precisely determine the submitochondrial localization of the three proteins (Fig. 1, rebuttal letter).*

*Finally, we discovered during the revision that we misinterpreted the experiment shown in Fig. 9D of the original manuscript. The band assigned to MaRF11-HA in Fig. 9D runs slightly faster (relative to the 15 kDa marker) than MaRF11-HA detected in Fig. 9C. Thus, we concluded that it corresponds to the unspecific band detected in Fig. 9C and not to MaRF11-HA. Reanalysis of the gel showed that the band corresponding to MaRF11-HA was of such low intensity that is was not possible to convincingly show its depletion upon TbPam16 RNAi. As a consequence, we had to remove Fig. 9D from the revised manuscript as well as all claims that binding of MaRF11 to TbPam16 protects it from proteasomal digestion (the last phrase in the abstract was modified and the last four lines in the result section were deleted). – We apologize for this oversight.*

----------------

**DETAILED POINT BY POINT RESPONSES TO THE REVIEWERS COMMENTS:**

**(Note: line numbers refer to the Manuscript file with highlighted changes)**

As you will see, the reviewers are generally in agreement that the study is interesting and well-conducted. However, they raise overlapping concerns that the model presented regarding the role of TbPAM16/18 in kDNA replication is too speculative at this stage and direct mechanistic evidence for the claims is lacking. Several experiments are proposed, including a control experiment to account for the potential indirect effects of altered import and direct biochemical evidence that TbMaRF11 acts in kDNA replication.

Based on the reviewer comments and following discussion with the Academic Editor, it is clear that a substantial amount of work would be required to meet address the concerns raised and make the study appropriate for PLOS Biology. However, given our and the reviewer interest in your study, we are happy to invite a thorough revision of the study that addresses all the reviewers' comments.

Specifically, we consider that the mechanistic aspects of the model should be addressed as the reviewers request.

*We have added the results of a new in vivo complementation experiment. It shows that a N-terminal myc-tag interferes with TbPam16 function, indicating that in addition to the integral membrane localization also the very N-terminus of the IMS-exposed 33 aa domain appears to be essential for TbPam16 function (revised Fig. 5, S6). This is consistent with our speculation that the TbPam16/TbPam18/MaRF11 complex might be part of a circuit that transmits a signal from the nucleus via the IMS to the matrix to regulate maxicircle replication.*

*We have now also added an additional SILAC RNAi analysis for MaRF11 and compared it with the SILAC RNAi datasets for TbPam18 and TbPam16 (Fig. S9, S10, S11) (see our response, entitled MaRF11 SILAC RNA ANALYSIS, to comment (a) reviewer 1). These results and the comparative analysis of the three SILAC RNAi datasets corroborates and extends the previous results we have obtained for MaRF11.*

We also agree with reviewer 4 that the lack of growth inhibition following RNAi of the PAM 16/18 in bloodstream forms is surprising, but not the major weakness of the paper which is focussed on a thorough mechanistic investigation of the procyclic forms. In some ways the blood stage forms are a distraction and the revision might be better spent confirming your model in procyclics.

*We did the requested experiment in bloodstream forms. The results are shown in newly added Fig. 4CD.*

Imaging and colocalization as suggested by reviewers 1 and 3 would be useful.

*As mentioned by the reviewers, knowing the precise submitochondrial localization of TbPam18, TbPam16 and MaRF11 might help in this matter. We have tried this extensively (see our response, entitled LOCALIZATION, to comment a) of reviewer 1).*

We share reviewer 2’s reservations about the presentation of the silac data, and think the data should be presented differently (more details about this at the end of the letter in "comments from the Academic Editor").

*For a more detailed analysis of the SILAC-RNAi data see 2^nd^ comment of reviewer 4.*

Reviewer 2's request of an analysis of maxi and minicircles in bloodstream forms following RNAi seems reasonable, although perhaps not strictly necessary to prove your model in procyclics.

*We did the requested experiment in bloodstream forms. The results are shown in newly added Fig. 4CD.*

We strongly agree with reviewer 4 that the evidence is consistent with the proposed model, but also with an indirect effect due to altered transport of mitochondrial proteins required for replication.

*We added new experiments showing that TbPam18 and TbPam16 are not involved in mitochondrial protein import. See new experiments and analyeis shown in new Figures S2, S3, S4, S9, S10 and S11. For a detailed discussion see 2^nd^ comment of reviewer 4*

Using the SILAC data to select a set of depleted proteins is a good suggestion for the proposed control experiment.

*We have now excluded that individual depletion of one of the most downregulated protein (TbTim54) in both the TbPam18 and TbPam16 RNAi cell lines could be responsible for the observed phenotype. See newly added experiment in new Fig. S4. For a detailed discussion see 2^nd^ comment of reviewer 4.*

**REVIEWS:**

**Reviewer #1:**

In this paper the authors show that in T. brucei procyclic forms, depleting PAM16 or PAM18 causes loss of maxicircles and release of minicircles from the kDNA network - presumably because they can't re-attach after replication. Oddly, the complex appears not to be required in bloodstream forms although there is no evidence that kDNA replication, per se, is any different from replication in procyclic forms. They demonstrate that the PAM16-PAM18 dimer is associated with a basic protein, MaRF11, depletion of which gives the same phenotype. There is also an association, of unknown significance, with the coenzyme Q complex. The transmembrane domains of PAM16 and PAM18 are required for function. The authors conclude:  "the two proteins have switched their function during evolution, from protein import to mitochondrial genome replication."  Since the functions of these proteins have not been examined in many different organisms, one could also consider the possibility that it's the other way around - that "the two proteins have switched their function during evolution, from mitochondrial genome replication to protein import."
The data are generally solid and support the direct conclusions.

Weaknesses are:
a) The precise roles of PAM16/PAM18/MaRF11 in kDNA network replication remain a mystery. The model in Fig 10, suggesting a role in nuclear-mitochondrial communication in kDNA replication control is wholly speculative.

*We agree that getting more insights into the mechanistic aspects of the proposed model would be important. We now present the results of a new in vivo complementation experiment. It shows that in addition to the integral membrane localization also the very N-terminus of the IMS-exposed 33 aa domain appears to be essential for TbPam16 function (revised Fig. 5, S6). This is consistent with our speculation that the TbPam16/TbPam18/MaRF11 complex might be part of a circuit that transmits a signal from the nucleus via the IMS to the matrix to regulate maxicircle replication.*

*A new paragraph describing these results has been added:*

*(lines 291-301)*

***“The IMS-exposed N-terminus of TbPam16 is functionally relevant***

*To find out, whether the very N-terminus of the 33 aa IMS-exposed domain is required for TbPam16 function we expressed N-terminally myc-tagged version of RNAi-resistant TbPam16 in the background of the TbPam16-RNAi cell line (Fig. 5A). As in the case of the ΔN-TbPam16-HA variant, the myc-TbPam16 was not able to complement growth (Fig. 5D). The myc-TbPam16 was highly expressed and a small amount was recovered in the crude mitochondrial fraction in a digitonin extraction. Moreover, essentially all of myc-TbPam16 in this fraction was recovered in the pellet after an alkaline carbonate extraction as would be expected for an integral membrane protein (Fig. S6B). These results suggest that the N-terminal myc-tag interferes with TbPam16 function indicating that at least part the IMS-exposed domain of TbPam16 is essential for its function.”*

I can only think of one simple experiment to examine this - immunofluorescence using the Pam16-myc cell line to see whether the complex is concentrated near the kinetoplast. (If so, this might support a mechanistic role.)

*We agree that, except for the localization (and the newly provided experiment we came up with focusing on the IMS domain which is described above), it is difficult to think of additional experiments, we have not already done that provide more mechanistic insights into our model. We put a lot of effort trying to localize TbPam18 and TbPam16 which is described in the next paragraph (entitled LOCALIZATION). - Moreover, we also provide a detailed SILAC RNAi analysis for MaRF11, which is discussed in the second paragraph of this section (entitled MaRF11 SILAC RNA ANALYSIS) that confirms and extends the previous results, we have obtained for MaRF11.*

*LOCALIZATION*

*As suggested by the reviewer, knowing the submitochondrial localization of MaRF11, TbPam16 and TbPam18 could be very informative to understand the roles of the three proteins. Thus, we invested a lot of time and effort to determine their precise localization. The results of our attempts to do so are summarized below:*

*MaRF11 is a soluble matrix protein and interacts with TbPam16. TbPam18 and TbPam16 are mitochondrial IM proteins and of all tagged variants of the two proteins only C-terminally tagged TbPam16 is functional ((1) and revised manuscript, Fig. 5C, S7). In case of the two Pam proteins, we therefore concentrated our efforts on TbPam16, also because we have an antiserum that recognizes untagged TbPam16.*

*The localization of MaRF11 and TbPam16 was investigated in three cell lines: the parental cell line 29-13 (29-13), 29-13 expressing MaRF11-HA (MaRF11-HA) and in 29-13 expressing MaRF11-HA which also allows Tet-inducible RNAi of TbHslV (MaRF11-HA in TbHslV RNAi). The latter was chosen because we have shown that MaRF11 accumulates in the cell upon TbHslV RNAi, which allows for better detection of the elusive protein (revised manuscript, Fig 9C ).* *Whole trypanosomes (WC) of all three cell lines were extracted with 0.5 % Triton. This allows the separation of a cytoskeletal fraction, containing subpellicular microtubules and flagella (P1), from a Triton-soluble fraction, comprising all cytosolic and Triton-soluble membrane proteins (SN1). Subsequently, P1 was treated with calcium which selectively dissolves the subpellicular microtubules and results in a final pellet corresponding to a crude flagellar fraction (Flag) (2). As shown previously, some of the isolated flagella in the Flag. fraction are still connected to the kDNA via the tripartite attachment complex (TAC) (3).*

*The results are shown in Fig 1 below. In the MaRF11-HA cell line essentially all MaRF11 is recovered in SN1 fraction, as expected for a matrix protein. In the MaRF11-HA/TbHslV-RNAi cell line, in which MaRF11-HA is accumulated, a significant fraction of the protein is recovered in the cytoskeletal fraction (P1) and a bit less in the crude flagellar fraction (Flag). In order to see whether the latter was bound to kDNA, we treated the Flag. fraction with DNase I. However, a stable interaction between MaRF11 and the kDNA could not be confirmed because the same amount of MaRF11 was recovered irrespectively of whether the kDNA was digested or not (Fig. 1B). It is possible that a fraction of MaRF11 is bound to the TAC, which would bring it into the vicinity of the kDNA, however alternative explanations are possible. The same fractions were also analyzed using the antiserum against TbPam16 (Fig. 1A). The immunoblot shows that the majority of TbPam16 was Triton-soluble, as expected for an integral IM protein. Only trace amounts of TbPam16 were detected in the P1 fraction and even less in the Flag. fraction.*

*Whole cells (WC), cytoskeletons (P1) and isolated flagella (Flag) were also analyzed by immunofluorescence microscopy. However, using our standard protocol, we were not able to detect any specific signal for MaRF11-HA or TbPam16(-HA). Unfortunately, the same was the case when combining immunofluorescence microscopy with a tyramide signal amplification protocol to increase the sensitivity of the analysis. This suggests that both ectopically expressed MaRF11-HA and TbPam16-HA as well as endogenous TbPam16 are expressed at a level too low to be detected by immunofluorescence microscopy even when using a signal enhancing protocol. This is unfortunate but in line with the observation that the three proteins are also difficult to detect in proteomics analyses ((1) and revised manuscript, Fig. 1).*

*In summary, while our experiments do not exclude that the three proteins are concentrated near the kDNA region, they also do not support such a localization. This is why we would like to provide the result in Fig. 1 for the reviewers only.*

***Figure 1*** *(for reviewers only)- Flagella extractions aiming to pinpoint the submitochondrial localization of MaRF11 and TbPam16.* ***(A)*** *29/13 parental cells, 29/13-based cells expressing MaRF11-HA and 29/13-based cells expressing MaRF11 and allowing for inducible RNAi against TbHslV were analyzed. Whole cells (WC) of all three cell lines were extracted with 0.5 % Triton. This allows the separation of a cytoskeletal fraction, containing subpellicular microtubules and flagella (P1), from a Triton-soluble fraction, comprising all cytosolic and Triton-soluble membrane proteins (SN1). Subsequently, P1 was treated with calcium which selectively dissolves the subpellicular microtubules and results in a final pellet corresponding to a crude flagellar fraction (Flag). All fractions were analyzed by immunoblot, which was stained with antisera against HA, TbPam16 and TAC40.* ***(B)*** *For the cell line expressing MaRF11-HA in TbHslV RNAi background, the same fractions as in (A) were generated again. Additionally, the Flag. fraction was treated with DNase I. Centrifugation after the DNase I treatment resulted in a supernatant (SN2), which contains previously DNA-bound proteins. The fractions were analyzed by immunoblot that was stained with anti-HA antibodies.*

*Recent spatial proteomics analyses of procyclic and bloodstream T. brucei cells allowed the assignment of the subcellular location of thousands of proteins based on the abundance distribution profiles that include 11 fractions each of three analyzed experiments (4). Intriguingly, the profiles of TbPam18, TbPam16 and MaRF11 were essentially identical, and coincided with marker proteins for the mitochondrial inner membrane. These results are consistent with our pulldown analysis of HA tagged TbPam16 and suggest that at steady state, in line with our model, all three proteins colocalize to the mitochondrial inner membrane.*

*We have replaced the final paragraph of the discussion section in which we extensively discuss the remaining open questions and limitations of our model, including our inability to precisely localize TbPam16, TbPam18 and MaRF11 by immunofluorescence. – Moreover, the new spatial proteomics paper which supports our results is cited in the same paragraph.*

*New paragraph focusing on the limitations of the proposed model*

*(lines 530-545)*

*“However, some aspects of the model remain speculative. For example, how MaRF11 controls maxicircle replication is still unknown. Its high pI would be consistent with a direct binding to the kDNA, but it is also possible that MaRF11 is only one component of a more elaborate maxicircle replication pathway that involves other factors further downstream. However, pulldown experiments aimed at identifying such factors in a cell line exclusively expressing tagged MaRF11 were not successful. Spatial proteomic analyses of the procyclic and bloodstream forms of T. brucei showed that at steady state TbPam18, TbPam16 and MaRF11 co-fractionate with IM marker proteins, consistent with the TbPam16 pulldown experiment (Fig. 7A). It would support our model if the three proteins were concentrated in the IM around the kDNA region. However, despite the use of signal enhancement protocols, we were unable to detect any of the three tagged proteins by immunofluorescence. This suggests that, consistent with our inability to detect any of the three proteins by MS in our global SILAC proteomics studies, they are of too low abundance to be detected by immunofluorescence. Despite these limitations the proposed model can serve as a guide for future experiments aimed at providing more insights into the intricate process of how the maxicircles and the kDNA in general are replicated.”*

*Finally, we would like to emphasize that our model (revised manuscript, Fig. 10) does not make stringent predictions regarding the localization of TbPam16 and TbPam18 other than that they should be IM proteins. The reason for this is that TbPam16, in addition to MaRF11, also interacts with components of the coenzyme Q biosynthetic complex, which likely is localized all over the IM (revised manuscript, Fig. 7A). Also, in the case of MaRF11 it is difficult to make a clear prediction where the protein should be localized. According to our model we would expect it to partially co-localize with TbPam16 as well as with the kDNA. However, its interaction with the kDNA might be transient and therefore difficult to detect.*

*MaRF11 SILAC RNA ANALYSIS*

*In addition to the SILAC RNAi analysis for TbPam18 and TbPam16, we now provide the same type of analysis for TbMaRF11 (Fig. S9, S10). This analysis corroborates and extends the previous results we have obtained for MaRF11. Together with quantitative correlation analysis of TbPam18, TbPam16, MaRF11 and TbTim17 SILAC RNAi datasets (Fig. S11), it strongly suggests that consistent with our model, TbPam18, TbPam16 and TbMaRF11, but not TbTim17, are involved in the same biological process.*

*The new analysis is discussed in a new paragraph of the revised manuscript:*

*(lines 387-409)*

*“Finally, we analyzed analyzed the Tb927.11.435 RNAi cell line using SILAC-based quantitative MS (Fig. S9 and S10), as was done for TbPam18 and TbPam16 RNAi cell lines (Fig. 1B, S2 and S3). 904 mitochondrial proteins were detected. The most affected proteins were MRPs and OXPHOS subunits, of which 58% and 17% were depleted more than 1.5-fold, making up for 60% of all 1.5-fold depleted mitochondrial proteins (Fig. S9). For seven protein categories consisting exclusively of nucleus-encoded proteins only two out of 205 protein were more than 1.5-fold depleted (Fig. S10). These results are very similar to what has been observed in TbPam18 and TbPam16 depleted cells (Fig. 1B, S2 and S3).*

*To quantify the similarities the depletion of TbPam18, TbPam16 and Tb927.11.435 caused on the mitochondrial proteome we performed a correlation analysis comparing all three datasets. Moreover, we included a previously published SILAC-RNAi dataset for TbTim17, the core component of the single trypanosomal TIM complex (Fig. S11). This allowed to compare how the mitochondrial proteome reacted to depletion of a general IM import factor versus factors involved in maxicircle replication. The results showed that the correlation factors among the TbPam18, TbPam16 and Tb927.11.435 datasets were between 0.69 and 0.73 (Fig. S11), which is very high considering the maximal possible correlation is 1.0. In contrast, the correlation factors of the TbTim17 dataset with the three other datasets were much lower ranging from 0.21 to 0.3 (Fig. S11).*

*These results show that depletion of TbPam18, TbPam16 and Tb927.11.435 caused the same proteomic phenotypes, strongly suggesting that the three proteins are involved in the same biological process. Moreover, the results also confirm that depletion of TbTim17, a general IM import factor, results in different changes to the mitochondrial proteome than were observed for the three other factors.”*

b) The lack of an effect of eliminating the complex in bloodstream forms is unexplained. The relevant paragraph in the discussion is unilluminating, saying merely (I paraphrase) that "control is different".

*It is true that we can presently not explain these findings, but as mentioned by reviewer 3 “…differential impact of a genetic ablation or a mutation in procyclic and bloodstream forms is not unprecedented.” - The flagellar connector which attaches the tip of the flagellum to the cell body can serve as an example, it is specific for procyclic cells (5). In contrast, in bloodstream forms the flagellum connects to the cell body via a different structure: the flagellar groove. Why the form of flagellar attachment differs between procyclic and bloodstream forms remains unexplained. – Given the differences in the dependence on kDNA-encoded proteins between procyclic and bloodstream forms it would not be surprising if the regulation of kDNA replication may also show differences.*

*We provide now additional experimental data showing that depletion of TbPam18 and TbPam16 in bloodstream forms do neither significantly affect kDNA disk size nor mini- or maxicircle abundance (newly added Fig. 4CD). - These results corroborate our unexpected findings.*

*New paragraph discussing the bloodstream form results:*

*(lines, 252-256)*

*“In line with the lack of growth phenotypes there was no significant change in the size of the kDNA networks (Fig. 4C) in neither the TbPam18-depleted RNAi cell line nor in the TbPam16 dKO cell line. Moreover, Southern blot analysis showed that the mean of mini- and maxicircle abundance was even higher in the two depleted cell lines, however this change was not statistically significant (Fig. 4D).”*

However I thought (maybe I'm wrong) that the major difference between bloodstream and procyclic form cycles are more in control of cytokinesis than of kDNA replication: procyclics can divide to make anucleate "zoids" containing just the kinetoplast, whereas bloodstream forms can't.

*We agree with the reviewer, but the difference in cytokinesis is already discussed in the manuscript.*

Suggested changes to the text:

The Introduction needs simple diagrams of the import complexes and the kDNA.

*These diagrams are now provided in new Fig. S1A and B.*

The Introduction needs to explain in detail the evidence that TbPam18 and TbPam16 are not required for protein import. This is really central to the start of the paper. (It's not possible to test mitochondrial import in dead parasites...)

*We added a new paragraph to the introduction explaining this in more detail (lines 71-79)*

*However, we do not understand the comment “It's not possible to test mitochondrial import in dead parasites...”. This is of course true, but we have a tight tetracycline-inducible RNAi system which allows to analyze genes/proteins that are essential (e.g. it is often possible to identify the biochemical defects that causes the growth arrest).*

The SILAC results need more discussion since most of the decreased proteins aren't mentioned at all.

*We agree with the comment. In the revised manuscript we have now analysed the SILAC RNAi data in more detail. Two new supplementary Figures (Fig. S2 and S3) show six panels each of both the TbPam16 and the TbPam18 RNAi cell lines filtered for seven further subgroups of mitochondrial proteins, none of which contains mitochondrially-encoded proteins. (The same analysis was also done for the newly added SILAC-RNAi dataset for MaRF11, Fig. S10 and S11)*

*The new analysis is discussed in a new paragraph of the revised manuscript:*

*(lines 157-173)*

*“In total, 67% (TbPam18) and 68% (TbPam16) of all more than 1.5-fold depleted mitochondrial proteins were either MRPs or subunits the OXPHOS complexes I, III, IV, and V, all of which contain mitochondrially encoded RNAs and/or proteins.*

*For protein categories consisting exclusively of nucleus-encoded proteins, the situation is very different. Neither of the 33 (TbPam18) and 30 (TbPam16) proteins detected in the category “kDNA replication factors” was more than 1.5-fold depleted (Fig. S2A and S3A). Essentially the same results are obtained for six other major previously defined subgroups of mitochondrial proteins, which exclusively consist of nucleus-encoded proteins (Fig. S2B-F and S3B-F). In all these subgroups combined, only three out of 279 detected proteins (TbPam18-RNAi) and not a single protein out of 274 (TbPam16-RNAi) are depleted more than 1.5-fold.*

*The observed phenotypes after depletion of TbPam18 and TbPam16 are notably different from RNAi cell lines targeting components of the protein import system, such as ATOM40 or TbTim17, in which depletion of members of all subgroups of mitochondrial proteins is observed. The SILAC RNAi results therefore confirm that TbPam18 and TbPam16 are not involved in mitochondrial protein import and suggest that the two proteins might be required for maintenance or replication of the kDNA.”*

In the supplementary tables, please include the most recent annotation, at least for the most reliably and strongly reduced proteins.

*The supplementary Tables have been modified accordingly*

It's notable that (applying strict filters) most of the decreased proteins are mitochondrial, and include various COX components.

*Yes indeed, this is discussed in the manuscript*

Why weren't the Pam proteins found in the RNAi SILAC?

*It is well known that not all proteins are detected with equal sensitivity in proteomic MS/MS analyses. All our data suggest that TbPam18 and TbPam16 are low abundant proteins. Moreover, they are small (TbPam16, 167 aa; TbPam18, 120 aa) resulting in fewer tryptic peptides. However, the two proteins can be detected in proteomic MS/MS analyses provided they are enriched in pull down experiments ((1) and revised manuscript, Fig. 7A).*

The supplementary tables need legends. (Easiest to put it on a separate sheet.)

*The supplementary tables have been modified accordingly*

The link with coenzyme Q needs to be discussed in a bit more detail, at least with a more precise description of what the Q complex does and also what, if anything, it might do in bloodstream forms. (Saying that CoQ is involved in "ubiquinone synthesis" is, to me at least, misleading - it doesn't make the whole molecule.)

*We have added more detailed information about COQ8 and COQ9 in the revised manuscript (lines 356-359).*

*The focus of our study is on procyclic cells. We therefore agree with the editor that “In some ways the blood stage forms are a distraction…” and would not like to discuss the role of coenzyme Q in the bloodstream form in the manuscript. - But we can provide the information for the reviewer. Based on a publication from 2014 (6) ubiquinone 9 (UQ9) is essential in bloodstream forms of T. brucei. In mitochondria UQ9 appears to be required for respiration via the trypanosomal alternative oxidase (TAO) and in glycosomes, where the regeneration of NADH depends on UQ9. Moreover, UQ9 likely is involved in the stabilization of the redox equilibrium.*

The fact that RNAi against COQ8 had no effect on kDNA is not completely conclusive, since the effect on protein levels was not measured.

*We do not understand the concern of the reviewer and are convinced that the experiment that RNAi-mediated depletion of COQ8 does not affect the kDNA is conclusive for the following reasons: COQ8 RNAi causes a growth retardation already after two days of induction. At that time point the COQ8 mRNA is not detectable anymore. We cannot measure COQ8 protein level as we do not have an antibody, however the observed growth retardation clearly indicates that the COQ8 protein is efficiently downregulated and that whatever small amount of the protein that might still be there is not enough to support growth. – We do not observe any change in the kDNA size even not two days into the growth phenotype demonstrating depletion of COQ8 while inhibiting growth, does not affect kDNA size.*

Could Pam18/16 nevertheless in some way be linked to the ubiquinone/ubiquinol redox cycle?

*Yes, this is indeed possible. However, in order to show this directly, further experimental analyses would be necessary, which would go beyond the scope of this work, which focuses on kDNA.*

Minor details (typos, grammar etc.)

Abstract:
I don't know what the authors mean by "MaRF11 directly mediates". It's the verb "mediates" that is the problem. For something to mediate we need to know what two things the protein is mediating between. Can the authors either be more specific, or choose a different wording?

*We agree that we do not have enough evidence to claim that MaRF11 is directly involved in maxicircle replication. – Thus, we have rewritten the conclusion section of the abstract.*

*(lines 36-40)*

*“Thus, we have discovered a protein complex, comprising TbPam18, TbPam16, and MaRF11, that controls maxicircle replication. We propose a working model, in which the matrix protein MaRF11 functions downstream of the two integral inner membrane proteins TbPam18 and TbPam16. Moreover, we suggest that the levels of MaRF11 are controlled by the mitochondrial proteasome.”*

Introduction

"The single mitochondrion of trypanosomes contains a single unit genome" .. This wording ("single unit") suggests that there is only a single genome, so it's confusing. How about turning the sentence around?
"The single mitochondrion of trypanosomes contains the most complex mitochondrial genome known in nature, called kinetoplast DNA."

*Since the phrase appears to be misleading we have corrected it as follows*

*(lines 90-92)*

*“The single mitochondrion of trypanosomes contains a single nucleoid containing all mitochondrial DNA. This DNA forms the most complex mitochondrial genome known in nature and is termed kinetoplast DNA (kDNA).”*

"(ca. 5000 copies, 1 kb each) that are arranged"  - "that" defines, "which" describes. So it should be "Which".

*Corrrected as suggested*

"The kDNA network consists to 90% of minicircles," Can't say "consist to". Try e.g. "minicircles comprise 90% of The kDNA network".

*Corrrected as suggested*

Results:

SILAC - I assume that both Pam16 and Pam18 were found in the uninduced controls?  If so, need to say this. Or if not, why not?

*No, the two proteins were not detected in the SILAC experiment. This is stated in the manuscript: “Surprisingly, neither TbPam18 nor TbPam16 were detected in the two SILAC RNAi experiments” (lines 137-138). – For possible reasons why the proteins are not detected see comment above.*

The percentages of reduced proteins are given so as to artificially inflate accuracy - there are fewer than 100 mitoribosomal or oxphos proteins, so giving percentages with decimal places makes no sense (you;d need 1000 to start with), Why not give the raw numbers instead.

*We now indicate both percentages and raw numbers in the revised manuscript (See Fig. 1, S2, S3, S10, S11)*

A general problem throughout the text: you describe what experiments were done in the past tense then suddenly shift - even mid-sentence - and give the results in the present tense. If experiments were done in the past, then the results were seen in the past too. Paragraph beginning: "To study the effects on the kDNA in more detail, we performed Southern blot analysis.."  suddenly changes tense in the middle (paraphrase - "DNA was extracted and maxicircles are decreased."). Other examples: "In the premix experiment, TbPam16 has been enriched...demonstrating that both CoIPs were successful." " This would be "DNA was extracted and maxicircles were decreased." and "TbPam16 was enriched...demonstrating that both CoIPs were successful." Similarly, "the amount of detected minicircles completely shifts to the supernatant" would be better as ""the detected minicircles completely shifted to the supernatant". Similarly "increase in the levels of MaRF11 for up to six days after
induction IS observed, whereas the levels of TbPam16 and EF1a REMAINED constant" - change "is" to "was". Many more examples.

*Corrected as suggested*

"we conclude that the function of TbPam18 is also redundant in the BSF."  - better to add the word "probably" here (probably redundant).

*Corrected as suggested*

For the Pam18 complementation, it's necessary to say that you do not know how much of the protein was produced in the complemented line, so the conclusion regarding the need for the TM domain has to be tentative. It's also necessary to say that the amounts of tagged Pam16s, relative to the amount of native protein, are unknown.

*We agree that this is true for TbPam18, but since tagged TbPam18 is not functional this is the best we can do. – However, the same is not the case for TbPam16. Here the HA-tagged version is functional. Because the immunoblots in Fig. S2B all stem from the same SDS-PAGE gel the expression levels of TbPam16-HA and ΔN-TbPam16-HA can directly be compared. This data is now provided in the new revised Fig. S6C. The result shows that ΔN-TbPam16-HA is approximately 1.5-fold overexpressed, indicating that insufficient expression of the TMD-lacking version of TbPam16 cannot explain the absence of complementation.*

Is the -TM version of Pam16 in the mitochondrion?

*Yes it is, see Fig. S6B*

"the same methods that were applied for TbPam16 and TbPam18" should either be "the same methods as were applied for TbPam16 and TbPam18" or simply ""the methods that were applied for TbPam16 and TbPam18".

*Corrected as suggested*

"One way of how TbPam18 and TbPam16 could regulate."  "of how" doesn't work. try replacing with "in which" or simply "that".

*Corrected as suggested*

"degradation, reminiscent to TbPIF2" - should be "reminiscent of TbPIF2"

*Corrected as suggested*

"a parallel reduction on the levels of .." should be "reduction in ..."

*Corrected as suggested*

**Reviewer #2:**

The manuscript submitted by Känel et al., titled " Evolutionary repurposing of trypanosomal Pam18 and Pam16 reveals a new regulatory circuit for mitochondrial genome replication" presents a well-executed study investigating the functions of TbPam18 and TbPam16 in trypanosomes. The authors effectively employ SILAC-based quantitative mass spectrometry to analyze RNAi-induced cell lines and uncover a significant impact on mitochondrial proteins, particularly mitoribosomal proteins (MRPs) and components of the oxidative phosphorylation (OXPHOS) pathway. The results, supported by various experimental techniques, reveal a link between the ablation of TbPam18 or TbPam16 and the depletion of maxicircles in kDNA, showing the essential role of these proteins in mitochondrial function. The manuscript effectively connects the observed effects on the mitochondrial proteome to the potential influence on maxicircle stability, showcasing a comprehensive understanding of the interplay between these integral IM proteins and mitochondrial function in trypanosomes. Immunofluorescence and Southern blotting is consistent with a role for TbPam16/18 in reattachment of minicircles. The identification of novel interaction partners Tb927.11.435 and TbCOQ8, and the involvement of the mitochondrial proteasome further informs the mechanism by which TbPam16/18 impact kDNA replication and opens paths for further research. The clarity of presentation, coupled with well-designed figures and comprehensive discussions, contributes to the overall strength of the manuscript.
Overall, the experiments are well-executed and support the conclusions, thereby contributing significantly to the understanding of TbPam18 and TbPam16 functions and the mechanism of kDNA replication in Trypanosoma brucei.  The major weakness lies in the studies on bloodstream forms (BF), which as presented are not definitive; additional Southern blot or qRT-PCR experiments should be performed to strengthen the conclusion that TbPam16/18 do not perform the same function in BF as they do in procyclic forms. Additionally, addressing the below comments would enrich the manuscript and provide a more comprehensive picture of the functional network involving TbPam18 and TbPam16 in trypanosomes throughout the life cycle.

Major comments (needs to be done)
1) With regard to BF parasites (Fig. 4), the authors conclude that Pam16/18 do not play the same role in BF as they do in procyclics.  However, it remains possible that a small kDNA replication defect might not lead to a growth defect in BF.  The authors need to at least analyze maxicircle abundance and minicircle release (as in Fig. 3) for the two BF cell lines to support the lack of any effect.

*We provide now additional experimental data showing that depletion of TbPam18 and TbPam16 in bloodstream forms do neither significantly affect kDNA disk size nor mini- or maxicircle abundance (newly added Fig. 4CD). - These results corroborate our unexpected findings.*

*New paragraph discussing the bloodstream form results:*

*(lines, 252-256)*

*“In line with the lack of growth phenotypes there was no significant change in the size of the kDNA networks (Fig. 4C) in neither the TbPam18-depleted RNAi cell line nor in the TbPam16 dKO cell line. Moreover, Southern blot analysis showed that the mean of mini- and maxicircle abundance was even higher in the two depleted cell lines, however this change was not statistically significant (Fig. 4D).”*

Minor comments (would strengthen the manuscript)
1) In Fig. 1A, the authors show the absence of TbPam16 in the TbPam18 RNAi line, suggesting a dependency of TbPam16 stability on TbPam18. However, the manuscript falls short in illustrating the impact of TbPam16 RNAi on TbPam18 stability. Creation of a cell line with tagged TbPam18 and TbPam16 RNAi would round out this story.

*We believe the suggested experiment would not be very informative because tagged TbPam18 is not functional.*

2) In the SILAC experiment in Fig. 1B, the authors state that "…58.6% and 62.5% were depleted more that 1.5 fold…".  Are these the percentages that also pass the p<0.05 cutoff or the total number regardless of significance?  Please clarify.

*It is the total number regardless of significance*

3) In Fig. 10, the model lacks influence as it primarily recapitulates the results without explaining the sequential order or direction of action of the proteins in wild type cells. A clearer demonstration of the order of events and the directionality involved is essential for a more compelling model.

*We agree and decided to remove the part that recapitulates the results. The revised Fig. 10 shows now only the working model and explicitly addresses its limitations.*

*We have also amended the text describing the model and limitations (last two paragraphs in the discussion section).*

4) Page 15 line 2; The sentence beginning "Whereas….." is not a complete sentence. please correct the language.

*Corrected as suggested*

**Reviewer #3:**

In this carefully executed study, von Känel et al. show that the inner membrane proteins TbPam18 and TbPam16, whose homologs regulate the pre-sequence translocase in most eucaryotes, are essential for maxicircle replication in the kinetoplast genome of procyclic (insect) developmental form of T. brucei. This is an intriguing connection, perhaps even a physical one, with the mitochondrial nucleoid as it connects through both mitochondrial membranes with the basal body of flagellum. It follows, that TbPam18 and TbPam16 anchoring in the inner membrane is critical for their function, which links maxicircle replication to the inner membrane. Along with their interactor discovered in this study, a positively charged protein MaRF11, these proteins join an exclusive club of factors essential for maxicircle, but not minicircle, replication. It is unclear whether these findings can be extended to the bloodstream form but the differential impact of a genetic ablation or a mutation in procyclic and bloodstream forms is not unprecedented.

*We agree with the Reviewer.* *- We have now also analyzed mini- and maxicircle abundance in bloodstream forms of the TbPam18 RNAi and the TbPam16 double knock out cell lines using DAPI-staining and Southern blots. Both cell lines grow normally (Fig. 4AB). We found no significant shrinkage of the kDNA disk in DAPI-stained cells after depletion of either of the two proteins (newly added Fig. 4C). Moreover, there was no significant change in the maxicircle and minicircle content when analyzed by Southern blots (newly added Fig. 4D). – These results corroborate our finding that the two proteins are only essential in the procyclic form.*

Although the experimental data are of high quality and generally support the most immediate conclusions, the model for a membrane-bound circuit that controls the replication (Fig. 10) is premature and too speculative. As written, this is a nice discovery work with several promising, but largely unsubstantiated mechanistic pitches.

*We agree that getting more insights into the mechanistic aspects of the proposed model would be important. We now present the results of a new in vivo complementation experiment. It shows that in addition to the integral membrane localization also the very N-terminus of the IMS-exposed 33 aa domain appears to be essential for TbPam16 function (revised Fig. 5, S6). This is consistent with our speculation that the TbPam16/TbPam18/MaRF11 complex might be part of a circuit that transmits a signal from the nucleus via the IMS to the matrix to regulate maxicircle replication.*

*A new paragraph describing these results has been added:*

*(lines 291-301)*

***“The IMS-exposed N-terminus of TbPam16 is functionally relevant***

*To find out, whether the very N-terminus of the 33 aa IMS-exposed domain is required for TbPam16 function we expressed N-terminally myc-tagged version of RNAi-resistant TbPam16 in the background of the TbPam16-RNAi cell line (Fig. 5A). As in the case of the ΔN-TbPam16-HA variant, the myc-TbPam16 was not able to complement growth (Fig. 5D). The myc-TbPam16 was highly expressed and a small amount was recovered in the crude mitochondrial fraction in a digitonin extraction. Moreover, essentially all of myc-TbPam16 in this fraction was recovered in the pellet after an alkaline carbonate extraction as would be expected for an integral membrane protein (Fig. S6B). These results suggest that the N-terminal myc-tag interferes with TbPam16 function indicating that at least part the IMS-exposed domain of TbPam16 is essential for its function.”*

The role of TbHslVU in regulating MaRF11 level would be one example that needs to be firmly established.

*Since only very little of MaRF11 is present in the cell and since we do not have antiserum against the protein. This is the best we can do.*

Finally, the authors may wish to consider analyzing submitochondrial localization and/or colocalization of TbPam18 TbPam16, MARF11. If their logic holds truth, it seems that these proteins should be concentrated in the IM adjacent to TAC/ basal body rather than distributed uniformly through the mitochondrial contour, as many IM proteins do.

*We put a lot of effort into these experiments but in the end could not really determine the submitochondrial localization of the two proteins. See comment a) reviewer 1 and Figure 1 in the rebuttal letter.*

**Reviewer #4:**

This is a very interesting, very well executed and described paper that explores the functions of the inner mitochondrial proteins TbPAM16 and TbPAM18 in T. brucei. Understanding these functions is of considerable interest because previous work has shown that, despite being orthologues of proteins in other eukaryotes, these factors are not part of the T. brucei PAM translocase, where their functions have been assumed by TbPAM27. Thus, the roles of TbPAM16 and TbPAM18 are mysterious. Through a very nice set of experiments, the authors demonstrate that loss of either protein leads to maxicircle kDNA (mitochondrial genome) loss and release of minicircles, and that their essentiality in procyclic form (PCF) parasites is dependent on their transmembrane domain. Moreover, they reveal interaction with several proteins, including TbMaRF11, whose loss also results in PCF-specific growth defects and kDNA loss, and whose abundance is under the control of the mitochondrial protease TbHsIVU.

From the above, exciting data the authors derive a model (Figure 10) in which TbPAM16, TbPAM18 and TbMaRF11 act in kDNA replication: TbMaRF11 acting directly, and TbPAM16/TbPAM18 by controlling TbMaRF11 proteosome-mediated degradation. While such a model would be very novel and interesting, unfortunately their data do not go far enough to prove it and it is therefore overly speculative.

*We agree that getting more insights into the mechanistic aspects of the proposed model would be important. We now present the results of a new in vivo complementation experiment. It shows that in addition to the integral membrane localization also the very N-terminus of the IMS-exposed 33 aa domain appears to be essential for TbPam16 function (revised Fig. 5, S6). This is consistent with our speculation that the TbPam16/TbPam18/MaRF11 complex might be part of a circuit that transmits a signal from the nucleus via the IMS to the matrix to regulate maxicircle replication.*

*A new paragraph describing these results has been added:*

*(lines 291-301)*

***“The IMS-exposed N-terminus of TbPam16 is functionally relevant***

*To find out, whether the very N-terminus of the 33 aa IMS-exposed domain is required for TbPam16 function we expressed N-terminally myc-tagged version of RNAi-resistant TbPam16 in the background of the TbPam16-RNAi cell line (Fig. 5A). As in the case of the ΔN-TbPam16-HA variant, the myc-TbPam16 was not able to complement growth (Fig. 5D). The myc-TbPam16 was highly expressed and a small amount was recovered in the crude mitochondrial fraction in a digitonin extraction. Moreover, essentially all of myc-TbPam16 in this fraction was recovered in the pellet after an alkaline carbonate extraction as would be expected for an integral membrane protein (Fig. S6B). These results suggest that the N-terminal myc-tag interferes with TbPam16 function indicating that at least part the IMS-exposed domain of TbPam16 is essential for its function.”*

There are two main problems that need to addressed.

First, it is clear that TbPAM16 and TbPAM18 are mitochondrial membrane proteins, and the authors' previous work indicates they are not part of a PAM translocase. However, is it truly unique that loss of TbPAM16 and TbPAM18, amongst membrane proteins, results in loss or perturbation of kDNA? A control experiment is needed to show that loss of such proteins does NOT result in kDNA perturbation.

*In the case of TbPam18 and TbPam16, appearance of small kDNAs can already clearly be seen at the timepoint the growth retardation becomes apparent. This is not the case for other inner membrane proteins as shown in previous publications. Ablation of TbTim17, TimRhom I and TimRhom II does not affect the kDNA at the time of the growth arrest, see supplementary Fig. 3 in reference (7). For TbTim17 this was confirmed independently in another study (8). The three proteins are essential for mitochondrial protein import, thus it may seem surprising that no effect on the kDNA is seen. The reason for this is that the cells stop growing well before the lack of import of kDNA replication factors can affect the kDNA. – Another, in our opinion very stringent control, is already shown in the original manuscript. Depletion of TbCOQ8, a subunit of the coenzyme Q biosynthetic complex, which is essential for normal growth, does not perturb the kDNA (Fig. 7 and 8) even though it interacts with TbPam16. – While we cannot test every inner membrane protein, these examples illustrate that depletion of an essential membrane protein does not by default affect the kDNA.*

It remains possible that TbPAM16 and TbPAM18 contribute in some way to protein import into the mitochondria and, indeed, the authors' SILAC proteome analyses (Fig.1) indicate changes in the abundance of many mitochondrial proteins. It is therefore possible that some of these proteins include factors needed for kDNA replication and the effects documented are an indirect result of altered import.

*We agree with the comment. In the revised manuscript we have now analysed the SILAC RNAi data in more detail. Two new supplementary Figures (Fig. S2A and S3) show six panels each of both the TbPam16 and the TbPam18 RNAi cell lines filtered for seven further subgroups of mitochondrial proteins, none of which contains mitochondrially-encoded proteins.*

*The new analysis is discussed in a new paragraph of the revised manuscript:*

*(lines 157-173)*

*“In total, 67% (TbPam18) and 68% (TbPam16) of all more than 1.5-fold depleted mitochondrial proteins were either MRPs or subunits the OXPHOS complexes I, III, IV, and V, all of which contain mitochondrially encoded RNAs and/or proteins.*

*For protein categories consisting exclusively of nucleus-encoded proteins, the situation is very different. Neither of the 33 (TbPam18) and 30 (TbPam16) proteins detected in the category “kDNA replication factors” was more than 1.5-fold depleted (Fig. S2A and S3A). Essentially the same results are obtained for six other major previously defined subgroups of mitochondrial proteins, which exclusively consist of nucleus-encoded proteins (Fig. S2B-F and S3B-F). In all these subgroups combined, only three out of 279 detected proteins (TbPam18-RNAi) and not a single protein out of 274 (TbPam16-RNAi) are depleted more than 1.5-fold.*

*The observed phenotypes after depletion of TbPam18 and TbPam16 are notably different from RNAi cell lines targeting components of the protein import system, such as ATOM40 or TbTim17, in which depletion of members of all subgroups of mitochondrial proteins is observed. The SILAC RNAi results therefore confirm that TbPam18 and TbPam16 are not involved in mitochondrial protein import and suggest that the two proteins might be required for maintenance or replication of the kDNA.”*

*These results contrast with the ones for proteins of the mitoribosome and the OXPHOS complexes I, III and IV (Fig. 1B), which contain core components that are encoded on the kDNA, explaining why a 66 and 67% of the subunits of these complexes get depleted more than 1.5-fold upon TbPam16 and TbPam18 RNAi.*

*The in-depth analysis of the SILAC RNAi data also revealed that a very efficiently downregulated protein, TbTim54, has previously been connected to mitochondrial protein import (9). In our initial analysis, we identified the protein by its open reading frame number and we did not realize it had been analysed before. We have now analysed TbTim54 in detail and show that its ablation does not cause kDNA loss.*

*The results are described in a new paragraph of the revised manuscript:*

*(lines 185-203)*

*However, there is an alternative explanation. The most depleted protein upon TbPam18 RNAi and the fourth most depleted one upon TbPam16 RNAi was TbTim54 (Fig. 1B). TbTim54 is unrelated to the yeast TIM22 complex subunit Tim54, but was proposed to mediate import of a subset of mitochondrial proteins with internal targeting sequences. Thus, we wanted to exclude that the loss of maxicircles observed in the TbPam18 and TbPam16 RNAi cell lines could be a consequence of the co-depletion of TbTim54 (Fig. 1B). Since RNAi depletion of TbTim54 did not affect growth, we produced a conditional double knock out cell line. In this cell line, depletion of the ectopically expressed copy of TbTim54 caused a growth arrest starting four days after tetracycline removal (Fig. S4A). However, even after six days of tetracycline removal, no shrinkage of the kDNA was observed (Fig. S4B). Thus, while TbTim54 is essential for normal growth, its depletion did not interfere with import of kDNA replication factors.*

*This is in line with the fact that even though TbTim54 has been reported to interact with TbTim17, it was not enriched in any of our previously published pulldowns of TIM complex subunits or associated proteins (TbTim17, TbTim42, ACAD, TbTim13, TbPam27). These results, together with the observation that TbPam18 and TbPam16 are neither associated with the single trypanosomal TIM complex nor involved in mitochondrial protein import (Fig 1B, S2 and S3) suggest that TbPam18 and TbPam16 are more directly involved in kDNA replication or maintenance.*

*Moreover we also provide a quantitative correlation analysis of the TbPam16, TbPam18 SILAC RNAi dataset and compare it to a previously published TbTim17 SILAC RNAi dataset (Fig. S11). (This analysis also includes the newly added SILAC RNAi dataset for MaR11, Fig. S9, S10). It shows that the TbPam16 and TbPam18 RNAi results strongly correlate with each other (corr. factor 0.73) but not with the one of TbTim17 (corr. factor 0.23-0.3) (Fig. S11). This essentially excludes that TbPam18 and TbPam16 function as general IM import factors.*

*The results (together with the new MaRF11 SILAC RNAi) are now described in the revised manuscript:*

*(lines 395-409, Fig. S11)*

*“To quantify the similarities the depletion of TbPam18, TbPam16 and Tb927.11.435 caused on the mitochondrial proteome, we performed a correlation analysis comparing all three datasets. Moreover, we included a previously published SILAC RNAi dataset for TbTim17, the core component of the single trypanosomal TIM complex (Fig. S11). This allowed to compare how the mitochondrial proteome reacted to depletion of a general IM import factor versus factors involved in maxicircle replication. The results showed that the correlation factors among the TbPam18, TbPam16 and Tb927.11.435 datasets were between 0.69 and 0.73 (Fig. S11), which is very high considering the maximal possible correlation is 1.0. In contrast, the correlation factors of the TbTim17 dataset with the three other datasets were much lower ranging from 0.21 to 0.3 (Fig. S11).*

*These results show that depletion of TbPam18, TbPam16 and Tb927.11.435 caused the same proteomic phenotypes, strongly suggesting that the three proteins are involved in the same biological process. Moreover, the results also confirm that depletion of TbTim17, a general IM import factor, results in different changes to the mitochondrial proteome than were observed for the three other factors.”*

*In summary, the RNAi results clearly show that neither TbPam16 nor TbPam18 are involved in mitochondrial protein import. Moreover, it is worth noting that the question whether or not TbPam16 and TbPam18 are involved in protein import was already the focus of a previous publication (1), a summary of which is now given in the revised introduction (lines 71-79).*

Second, the authors propose (in many places, including Fig.10) that TbMaRF11 acts DIRECTLY in kDNA replication. This role and connection to TbPAM16 and TbPAM18 is crucial for the model proposed, but no evidence for a function of TbMaRF11 in kDNA replication, or indeed for localisation to the kDNA is provided. Without any such biochemical or imaging analysis, we cannot discount an indirect role, such as in some form of import, acting with TbPAM16 and TbPAM18.

*We agree and provide a revised simplified version of Fig. 10 which shows now only the working model and explicitly addresses its limitations.*

*We have also amended the text describing the model as well as its limitations:*

*(lines: 516-545)*

*“Based on the results described above, we propose the following working model for a mitochondrial inner membrane-bound protein complex involved in the replication of the maxicircle portion of the kDNA (Fig. 10). The complex includes the soluble protein MaRF11 and the integral IM proteins TbPam18 and TbPam16. The latter two probably form a heterodimer and have their J-like domains exposed to the matrix. All three proteins are individually essential for maxicircle replication in procyclic trypanosomes. The fact that MaRF11 is a soluble matrix protein suggests that it may function downstream of the integral IM proteins TbPam18 and TbPam16. Furthermore, we have shown that MaRF11 levels are controlled by proteolytic degradation by TbHslVU.*

*In T. brucei, the mitochondrial S-phase is precisely coordinated with the nuclear S-phase by an as yet unknown signal. Since not only the TMDs of TbPam18 and TbPam16 but also the IMS-exposed N-terminus of TbPam16 are essential for normal growth, it is tempting to speculate that the TbPam18/TbPam16/MaRF11 complex may play a role in this coordination.*

*Some aspects of the model remain speculative. For example, how MaRF11 controls maxicircle replication is still unknown. Its high pI would be consistent with a direct binding to the kDNA, but it is also possible that MaRF11 is only one component of a more elaborate maxicircle replication pathway that involves other factors further downstream. However, pulldown experiments aimed at identifying such factors in a cell line exclusively expressing tagged MaRF11 were not successful. Spatial proteomic analyses of the procyclic and bloodstream forms of T. brucei showed that at steady state TbPam18, TbPam16 and MaRF11 co-fractionate with IM marker proteins, consistent with the TbPam16 pulldown experiment (Fig. 7A). It would support our model if the three proteins were concentrated in the IM around the kDNA region. However, despite the use of signal enhancement protocols, we were unable to detect any of the three tagged proteins by immunofluorescence. This suggests that, consistent with our inability to detect any of the three proteins by MS in our global SILAC proteomics studies, they are of too low abundance to be detected by immunofluorescence. Despite these limitations the proposed model can serve as a guide for future experiments aimed at providing more insights into the intricate process of how the maxicircles and the kDNA in general are replicated.”*

*We have tried to localize the proteins without much success (see our response, entitled LOCALIZATION, to comment a) of reviewer 1). However, we now provide further analyses which essentially exclude that TbPam18, TbPam16 or MaRF11 are involved in mitochondrial protein import (see comments above).*

Minor issues:
1. Page 6, the RNAi experiments do not allow us to conclude that TbPam16 and TbPam18'form a heterodimer as in yeast'.

*Corrected*

2. Page 6. The statement, 'Taken together, the that the ablations of TbPam18 and TbPam16 could affect the kDNA' is premature; this conclusion can only be reached by experiments detailed later.

*The whole paragraph has been modified.*

3. Page 8, 'we conclude that the function of TbPam18 is also redundant in the BSF'; why do they reach this conclusion, given the inability to generate a null mutant?

*Since RNAi cell line of TbPam18 and TbPam16 in bloodstream forms behave the same, we suggest that our inability to create a conditional null mutant is a technical and not a biological problem.*

4. Page 11, The statement 'Furthermore, this also holds true for RNAi-induced knockdown of MaRF11 in BSF NYsm cells, which does not cause a change in the growth rate', should be reworded as it appears to infer that BSF RNAi results in kDNA loss, which has not been documented.

*The phrase has been modified.*

5. Do the essentially negative results in the section 'The J-domain of yeast Pam18 cannot complement the loss of the TbPam18 J-like domain' add anything to the paper?

*We believe it does. TbPam18 is a homologue of yeast Pam which however, as shown in our study, has a different function than the yeast protein, which is consistent with the observed results.*

Additional comments from the Academic Editor

I find the silac experiment a bit difficult to interpret because it seems to lack a control analysis, ie its a mitochondrial enriched fraction and the two sets of proteins encoded on kDNA are depleted but they need to be compared to a set of mitochondrial proteins not encoded on kDNA to conclude that the kDNA encoded genesets are depleted above background depletion of mitochondrial proteins.

*We agree with the comment. In the revised manuscript we have now analysed the SILAC RNAi data in more detail. Two new supplementary Figures (Fig. S2 and S3) show six panels each, of both the TbPam16 and the TbPam18 RNAi cell lines filtered for seven further subgroups of mitochondrial proteins, none of which contains mitochondrially-encoded proteins (for a detailed discussion see above)*

In general abbreviations used infrequently should be replaced with expanded text to aid readibility, there is no word limit in PLOS Biology so no need for abbreviations. Fig 2 a and b tet (d) should be replaced with days as in the graphs…

*All Figures have been edited accordingly: “d” has been replaced by “days”.*

…further examples include PCF, BSF, OXPHOS, PAM, IM

*We disagree on this point. These abbreviations are used frequently (BSF, 22 times; PCF, 13 times; OXPHOS, 17 times). PAM also appears in the gene names TbPam16 etc. We do not think it would make the text more readable if we replace these abbreviations but rather more clumsy, because the mentioned abbreviations would need to be replaced with 2 words each, or in the case of PAM even with four words.*

Page 8 “Integral membrane localization of TbPam18 and TbPam16 is functionally relevant”. These experiments need to be more clearly explained, preferably in results but could also be in the methods. I assume the tet induces the RNAi but the complementation is also simultaneously induced,…

*Yes, RNAi and the complementation are induced at the same time. This has been clarified now. - Two phrases have been added to the paragraph in question:*

*(lines 268-271)*

*“This was achieved by ectopically expressing the RNAi-resistant variants of the two proteins under tetracycline control in the background of the corresponding RNAi cell lines. In the complemented cell lines tetracycline addition therefore simultaneously induces RNAi and expression of the RNAi resistant varaints of the two proteins.”*

…it was unclear to me why the complementation was induced by tet instead of being constitutively expressed from the puromycin maintained ectopic plasmid.

*Surely constitutive expression would also work. However, we generally use ectopic expression because in our hands its technically easier and works just as well. This is especially the case when synthetic RNAi-resistant versions of the genes are used.*

Page 12 “Moreover, expression of HA-tagged MaRF11 in the induced TbPam16 RNAi cell line causes a parallel reduction on the levels of TbPam16 and MaRF11, respectively (Fig. 9D).”
I think this sentence is incorrect, the pam16 is reduced because of RNAi not because tagged marf11 is expressed.

*The paragraph has been removed as a whole (see above).*

***References***

*1.	von Känel C, Muñoz-Gómez SA, Oeljeklaus S, Wenger C, Warscheid B, Wideman JG, et al. Homologue replacement in the import motor of the mitochondrial inner membrane of trypanosomes. eLIFE. 2020;9: e52560.*

*2.	Sasse R, Gull K. Tubulin posttranslational modifications and the construction of microtubular organelles in Trypanosoma brucei. J Cell Sci. 1988;90:577-89.*

*3.	Schnarwiler F, Niemann M, Doiron N, Harsman A, Kaser S, Mani J, et al. Trypanosomal TAC40 constitutes a novel subclass of mitochondrial beta-barrel proteins specialized in mitochondrial genome inheritance. Proc Natl Acad Sci U S A. 2014;111(21):7624-9.*

*4.	Moloney NM, Barylyuk K, Tromer E, Crook OM, Breckels LM, Lilley KS, et al. Mapping diversity in African trypanosomes using high resolution spatial proteomics. Nat Commun. 2023;14(1):4401.*

*5.	Varga V, Moreira-Leite F, Portman N, Gull K. Protein diversity in discrete structures at the distal tip of the trypanosome flagellum. Proc Natl Acad Sci U S A. 2017;114(32):E6546-E55.*

*6.	Lai DH, Poropat E, Pravia C, Landoni M, Couto AS, Rojo FG, et al. Solanesyl diphosphate synthase, an enzyme of the ubiquinone synthetic pathway, is required throughout the life cycle of Trypanosoma brucei. Eukaryot Cell. 2014;13(2):320-8.*

*7.	Harsman A, Oeljeklaus S, Wenger C, Huot JL, Warscheid B, Schneider A. The non-canonical mitochondrial inner membrane presequence translocase of trypanosomatids contains two essential rhomboid-like proteins. Nat Commun. 2016;7:13707.*

*8.	Tyc J, Klingbeil MM, Lukes J. Mitochondrial heat shock protein machinery hsp70/hsp40 is indispensable for proper mitochondrial DNA maintenance and replication. MBio. 2015;6(1).*

*9.	Singha UK, Tripathi A, Smith JT, Jr., Quinones L, Saha A, Singha T, et al. Novel IM-associated protein Tim54 plays a role in the mitochondrial import of internal signal-containing proteins in Trypanosoma brucei. Biol Cell. 2021;113(1):39-57.*
